# Supplementary material for: Harnessing Greenhouse Gases Absorption by Doped Fullerenes with Externally Oriented Electric Field
Source: Molecules. 2022 May 6;27(9):2968. doi: 10.3390/molecules27092968 (PMC9105219; doi:10.3390/molecules27092968)

Supporting Information for

Harnessing Greenhouse Gases Absorption by Doped Fullerenes with Externally Oriented Electric Field

Rodrigo A. Lemos Silva [1,*], Daniel F. Scalabrini Machado [2], Núbia Maria Nunes Rodrigues [3],

Heibbe C. B. de Oliveira [4], Luciano Ribeiro [3] and Demétrio A. da Silva Filho [1,*]

[1]Institute of Physics, University of Brasília, 70.919-970, Brasília, Brazil

[2]Laboratório de Modelagem de Sistemas Complexos (LMSC), Instituto de Química, Universidade de Brasília, 70919-970, Brasília, Brazil

[3]Laboratório de Estrutura Eletrônica e Dinâmica Molecular (LEEDMOL), Instituto de Química, Universidade Federal de Goiás, Goiânia, Brazil

[4]Grupo de Química Teórica e Estrutural de Anápolis, Campus de Ciências Exatas de Anápolis, Universidade Estadual de Goiás, Anápolis, Brazild

Correspondence: silvarodrigo021@gmail.com (R.A.L.S.); dasf@unb.br* (D.A.d.S.F.)

Contents

Figure S1. Orientation of the electric field vector with respect to the $C_{19}Si$ cage............. 3

Table S1. Optimized coordinates for C_{20} and $C_{19}Si$ fullerenes at theoretical levels ωB97XD/6-31G(d) and ωB97XD/6-311+G(d,p). Electic field F in atomic units and Total energies in hartree. 3

Table S2. Optimized coordinates for the dimers formed by pure and doped fullerenes and CO, CO_2 and N_2 gases in each of the studied field values. Electic field F in atomic units and BSSE-corrected adsorption energies in electron-volts. 4

Table S3. Frontier orbital energies (in eV) for C_{20} and $C_{19}Si$ fullerenes. Percentual increase of the HOMO-LUMO gap, ΔE_{HL} is also shown. 16

Table S4. Si-C bond lengths and Merz-Kollmann (MK) charges on the Si-atom and adjacent carbons. 17

Table S5. Adsorption energies (in eV), E_{ads}, and adsorption energy with and without BSSE corrections, $E_{ads}(BSSE)$, for the dimers formed between C_{20} fullerenes with CO, CO_2 and N_2 molecules calculated with the theoretical levels ωB97XD /6-31G(d) and ωB97XD/6-311+G(d,p). 17

Table S6. Externally oriented electric field effect on the BSSE correction, adsorption energies, E_{ads}, and BSSE corrected, $E_{ads}(BSSE)$, for the interaction of C_{20} and $C_{19}Si$

fullerenes with the CO, CO_2 and N_2 molecules as obtained at the ωB97XD/6-31G(d) and ωB97XD/6-311+G(d,p) levels of theory. Adjusted adsorption energy, E_{ads}(fit) and the R-squared coefficient of determination, R^2, are also presented. All energies in eV. 18

Table S7. Topological parameters calculated using the Quantum Theory of Atoms in Molecules (QTAIM) with the theoretical level ωB97XD /6-31G(d). Results are presented for all dimers formed by C_{20} and $C_{19}Si$ fullerenes with adsorbed gas molecules. The results were obtained for all electric field values and are all presented in atomic units. 20

Table S8. Topological parameters calculated using the Quantum Theory of Atoms in Molecules (QTAIM) with the theoretical level ωB97XD /6-311+G(d,p). Results are presented for all dimers formed by C_{20} and $C_{19}Si$ fullerenes with adsorbed gas molecules. The results were obtained for all electric field values and are all presented in atomic units. 22

Table S9. Critical bond points, BCP, isosurfaces and scatter plots for the dimers formed by C_{20} and $C_{19}Si$ fullerenes with the adsorbed gas molecules. The results were obtained for all electric field values using both the theoretical level ωB97XD/6-31G(d) and ωB97XD/6-311+G(d,p). 23

Table S10. Energies (in eV) of the HOMO, E_H, LUMO, E_L and the gap between them, E_{HL}, calculated with the theoretical levels ωB97XD/6-31G(d) and ωB97XD/6-311+G(d,p), for the dimers. The percentual change in the fullerene energy gap after adsorbing the gas, in relation to the fullerene energy gap before adsorption, is represented by ΔE_{HL} (in %). Positive values of ΔE_{HL} indicate an increase in the gap, negative values indicate a reduction. 19

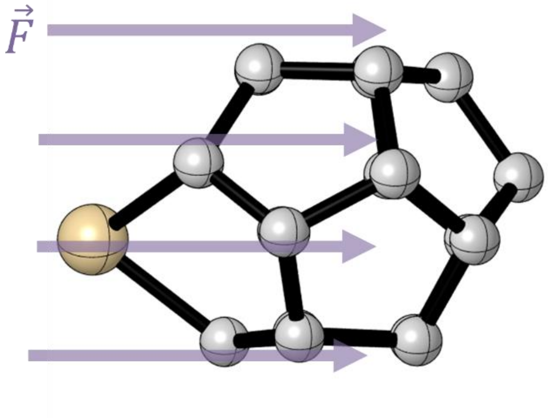

Figure S1. Orientation of the electric field vector with respect to the $C_{19}Si$ cage.

Table S 1. Optimized coordinates for C_{20} and $C_{19}Si$ fullerenes at theoretical levels ωB97XD/6-31G(d) and ωB97XD/6-311+G(d,p). Electic field F in atomic units and Total energies in hartree.

**ωB97XD/6-31G(d)**				**ωB97XD/6-311+G(d,p)**			
**C_{20}**							
$F = 0.000$ $Energy = -761.177568$				$F = 0.000$ $Energy = -761.327563$			
C	1.96817100	-0.00013300	-0.69597200	C	1.96549500	-0.00012400	-0.69506900
C	1.17416600	-1.17719400	-1.11374100	C	1.17269000	-1.17611700	-1.11335400
C	1.96818000	0.00019900	0.69595700	C	1.96550400	0.00019000	0.69505300
C	1.17417700	-1.17663900	1.11421000	C	1.17268800	-1.17556800	1.11383100
C	0.76585100	-1.94678400	0.00041000	C	0.76534100	-1.94358200	0.00041000
C	1.17413600	1.17723200	1.11373200	C	1.17266000	1.17615500	1.11334400
C	-0.00000400	0.71876000	1.80649800	C	-0.00000400	0.71793600	1.80537200
C	0.00002100	-0.71792800	1.80682100	C	0.00002000	-0.71709800	1.80568900
C	0.76578600	1.94680900	-0.00041700	C	0.76527500	1.94360700	-0.00041600
C	-0.76585100	1.94678400	-0.00041000	C	-0.76534100	1.94358200	-0.00041000
C	-1.17416600	1.17719400	1.11374100	C	-1.17269000	1.17611700	1.11335400
C	1.17412600	1.17667800	-1.11422100	C	1.17263900	1.17560800	-1.11384100
C	-0.00002100	0.71792800	-1.80682100	C	-0.00002000	0.71709800	-1.80568900
C	-1.17417700	1.17663900	-1.11421000	C	-1.17268800	1.17556800	-1.11383100
C	0.00000400	-0.71876000	-1.80649800	C	0.00000400	-0.71793600	-1.80537200
C	-1.17413600	-1.17723200	-1.11373200	C	-1.17266000	-1.17615500	-1.11334400
C	-1.96818000	-0.00019900	-0.69595700	C	-1.96550400	-0.00019000	-0.69505300
C	-1.96817000	0.00013300	0.69597200	C	-1.96549500	0.00012400	0.69506900
C	-1.17412600	-1.17667800	1.11422100	C	-1.17263900	-1.17560800	1.11384100
C	-0.76578600	-1.94680900	0.00041700	C	-0.76527500	-1.94360700	0.00041600
**$C_{19}Si$**							
$F = 0.000\ a.u.$ $Energy = -1012.776536$				$F = 0.000\ a.u.$ $Energy = -1012.776536$			
C	0.91421500	-1.35551500	1.29928900	C	0.91350600	-1.84301600	0.34754400
C	-0.46189100	-1.63458100	0.89484300	C	-0.46149100	-1.85569100	-0.14347700
C	0.91450100	-0.06538400	1.87665100	C	0.91394900	-1.07876600	1.53446200
C	-0.46174800	0.42245100	1.81454000	C	-0.46096700	-0.63649900	1.74873400
C	-1.34175300	-0.58521000	1.30795100	C	-1.33934700	-1.20253900	0.77463600
C	1.74935900	0.76130800	1.05169500	C	1.74766000	0.06292500	1.29723800

C	0.91452400	1.80318500	0.52415000	C	0.91405800	1.22253600	1.42223800
C	-0.46154200	1.59262900	0.96830600	C	-0.46085100	0.80361500	1.67892200
C	2.40815000	0.00005600	-0.00001200	C	2.40412100	-0.00015800	-0.00004700
C	1.74933200	0.53028200	-1.18500300	C	1.74775700	1.09179100	-0.70348400
C	0.91451600	1.65802200	-0.88145600	C	0.91411000	1.86820400	0.16696600
C	1.74918000	-1.29269300	0.13354700	C	1.74732600	-1.15578700	-0.59420500
C	0.91418900	-1.59240900	-0.99460900	C	0.91351100	-0.78957200	-1.70099700
C	0.91484500	-0.44771000	-1.82373900	C	0.91405500	0.62050800	-1.77010800
C	-0.46181500	-1.78319500	-0.54180800	C	-0.46143000	-1.19624900	-1.42584500
C	-1.34137600	-0.84011300	-1.16072200	C	-1.33919400	-0.06934500	-1.42861400
C	-0.46141100	0.04244100	-1.86261100	C	-0.46083400	1.05213400	-1.53507800
C	-0.46148600	1.36092700	-1.27331800	C	-0.46078800	1.83309200	-0.32315800
C	-1.34103000	1.42507400	-0.14712300	C	-1.33885800	1.27204300	0.65410100
Si	-2.72089700	0.00018600	-0.00024400	Si	-2.71984000	0.00033200	0.00007400

Table S2. Optimized coordinates for the dimers formed by pure and doped fullerenes and CO, CO_2 and N_2 gases in each of the studied field values. Electic field F in atomic units and BSSE-corrected adsorption energies in electron-volts.

**ωB97XD/6-31G(d)**				**ωB97XD/6-311+G(d,p)**			
**C_{20}-CO**							
$F = 0.000$ $E_{ads}(BSSE) = -0.04027087$				$F = 0.000$ $E_{ads}(BSSE) = -0.04215341$			
O	3.34694300	-1.80506800	0.01811600	O	3.31257900	-1.80409600	-0.00638300
C	2.84804700	-0.78483600	0.01561800	C	2.81785900	-0.79251200	-0.01452300
C	7.10590900	1.54087900	-1.04107100	C	7.10338800	1.53702400	-1.03496000
C	8.15189300	0.88878000	-1.78265500	C	8.14497600	0.88517100	-1.78004600
C	7.68118100	2.17225900	0.08656100	C	7.68248900	2.16892800	0.08883400
C	9.18190700	1.87216300	0.04331300	C	9.18249800	1.87070500	0.03835600
C	9.40715300	1.08075200	-1.10748700	C	9.40199500	1.07971400	-1.11231000
C	7.15262400	1.45547100	1.18492300	C	7.16149800	1.45667800	1.19085900
C	8.22795100	0.74904100	1.82827900	C	8.23985000	0.75204300	1.82799100
C	9.45442200	0.99510700	1.11853300	C	9.46152700	0.99890800	1.11321900
C	6.13412700	0.47380400	0.74935500	C	6.14309600	0.47282300	0.76014300
C	6.68897000	-0.85068600	1.10429100	C	6.70056200	-0.84895500	1.11459200
C	7.94490600	-0.65854600	1.77880900	C	7.95835300	-0.65429300	1.78176100
C	6.10467400	0.52802400	-0.64163500	C	6.10638300	0.52492400	-0.62850900
C	6.64176400	-0.76501000	-1.12208600	C	6.64108600	-0.76808600	-1.11135600
C	6.91461300	-1.64107000	-0.04664800	C	6.92053300	-1.63887000	-0.03623700
C	7.86888600	-0.51885300	-1.83116300	C	7.86347500	-0.52121200	-1.82547600
C	8.94380100	-1.22584200	-1.18837800	C	8.94131100	-1.22636600	-1.18876800
C	8.41492500	-1.94243400	-0.08994000	C	8.42004700	-1.93856800	-0.08673800
C	8.99049600	-1.31120700	1.03774600	C	8.99942000	-1.30661200	1.03706300
C	9.99192700	-0.29814000	0.63786700	C	9.99668100	-0.29435500	0.63014900
C	9.96248900	-0.24398100	-0.75235100	C	9.96000400	-0.24238700	-0.75766300
**C_{20}-C0_2**							
$F = 0.000$ $E_{ads}(BSSE) = -0.06556463$				$F = 0.000$ $E_{ads}(BSSE) = -0.07502922$			
C	0.58561400	0.04675200	-0.41514800	C	0.62769000	0.12142600	-0.49236200
O	0.65222200	-0.11126900	0.73682800	O	0.75707800	-0.23508500	0.60113400
O	0.51242700	0.20215400	-1.56707300	O	0.49117600	0.47711300	-1.58391000

C	5.09326500	1.57819800	-0.99626800	C	5.00397000	1.52753400	-1.00164100
C	5.80464400	0.60183500	-1.77425100	C	5.71549600	0.55341100	-1.78089600
C	5.95372500	2.06216100	0.02663500	C	5.90262700	2.05078800	0.04246900
C	7.27627200	1.30785900	-0.11891200	C	7.16704700	1.29046200	-0.10866300
C	7.12118600	0.42514100	-1.21987300	C	7.03427400	0.40510400	-1.23268600
C	5.35427900	1.61579500	1.21969700	C	5.35171100	1.66819300	1.26010000
C	6.22410900	0.63410500	1.81685000	C	6.27427100	0.67467200	1.83967500
C	7.38779700	0.45057200	0.99445500	C	7.42958600	0.51335000	1.03799800
C	4.04978100	0.96293300	0.93543900	C	4.11288600	0.91728400	0.94599300
C	4.20411500	-0.43844000	1.34568000	C	4.27788600	-0.44201700	1.38186500
C	5.51978100	-0.61591700	1.89887700	C	5.59670100	-0.58935000	1.92711900
C	3.87380900	0.96468100	-0.44627600	C	3.85687900	0.91448400	-0.44060900
C	3.93751600	-0.46425400	-0.86908600	C	3.88001800	-0.55067700	-0.89125700
C	4.04882400	-1.32110100	0.24313600	C	4.14760100	-1.32531200	0.25607900
C	5.10192200	-0.64775400	-1.69253700	C	5.03865800	-0.70987900	-1.69043600
C	5.97097300	-1.62925700	-1.09523500	C	5.96079200	-1.70393100	-1.11290200
C	5.37045100	-2.07664900	0.09669500	C	5.40990900	-2.08744500	0.10441900
C	6.23055200	-1.59222600	1.12033500	C	6.30910300	-1.56572100	1.14991700
C	7.44906800	-0.97744000	0.57078000	C	7.45273600	-0.94965300	0.58800900
C	7.27766900	-0.97788200	-0.81074600	C	7.20190500	-0.95475800	-0.79940900
**C_{20}-N_2**							
$F = 0.000$				$F = 0.000$			
$E_{ads}(BSSE) = -0.04191301$				$E_{ads}(BSSE) = -0.04214772$			
N	0.58478100	-1.97944200	-0.13254500	N	0.41249400	-1.57070000	-0.52204300
N	0.22177400	-0.96292700	0.08912200	N	0.26623900	-1.40781400	0.54810000
C	4.40572800	1.54579000	-1.08795800	C	4.42922300	1.53961000	-1.11932400
C	5.43565800	0.83559500	-1.79713600	C	5.46409300	0.81720000	-1.80600800
C	4.99058300	2.18474400	0.03067400	C	5.00261600	2.18398500	-0.00206400
C	6.48036700	1.83084800	0.01615600	C	6.49079500	1.82649200	0.00383600
C	6.68852900	0.99870000	-1.10939100	C	6.70972700	0.98594100	-1.10919600
C	4.42075000	1.52060900	1.14085000	C	4.41997700	1.53416600	1.10964100
C	5.46059400	0.79227800	1.81698700	C	5.44849600	0.81164400	1.80531900
C	6.70466700	0.97306900	1.11750000	C	6.69886200	0.98085900	1.11758200
C	3.37182400	0.56522500	0.71648000	C	3.37393700	0.58001300	0.68591700
C	3.87209800	-0.76789100	1.11391300	C	3.86402300	-0.75134700	1.10645200
C	5.12541800	-0.60470100	1.80109900	C	5.10978200	-0.58293000	1.80343500
C	3.36178600	0.58242300	-0.67534600	C	3.37963000	0.58144200	-0.70384900
C	3.85604200	-0.74219700	-1.11333800	C	3.87486400	-0.74622700	-1.12013500
C	4.08070600	-1.59950600	-0.01180600	C	4.08258200	-1.59213100	-0.00644600
C	5.10045000	-0.56139500	-1.81229800	C	5.12527800	-0.57739200	-1.80795000
C	6.13996400	-1.29024100	-1.13648600	C	6.15336100	-1.30002500	-1.11208200
C	5.56995800	-1.95434700	-0.02629900	C	5.57063700	-1.95000100	-0.00048500
C	6.15504600	-1.31547200	1.09238600	C	6.14420400	-1.30545700	1.11664600
C	7.19898300	-0.35211500	0.67951700	C	7.19418600	-0.34733900	0.70102400
C	7.18908200	-0.33498300	-0.71208300	C	7.19978200	-0.34592700	-0.68837000
**C_{19}Si-CO**							
$F = 0.000$				$F = 0.000$			
$E_{ads}(BSSE) = -0.70264080$				$E_{ads}(BSSE) = -0.73799673$			
O	2.01502800	-0.01037600	-0.00025500	O	2.01053700	-0.01993600	0.00877900
C	3.14119300	-0.01035200	0.00416500	C	3.12664200	-0.01236700	0.02139200
C	8.18173800	-1.37815000	1.29081900	C	8.18322500	-1.37772500	1.29143500
C	6.84077900	-1.70008500	0.93491700	C	6.84358000	-1.69783900	0.93565400

C	8.17811900	-0.09251600	1.86489000	C	8.18137600	-0.09268300	1.86216600
C	6.80722200	0.39724100	1.83179100	C	6.81098700	0.39819100	1.83171700
C	5.91034300	-0.66680100	1.53729200	C	5.91495700	-0.66098500	1.53607100
C	8.99384900	0.72449500	0.99628100	C	8.99552200	0.72393400	0.99540100
C	8.17088900	1.80029200	0.49388800	C	8.17432400	1.79810700	0.49239500
C	6.80234500	1.60888300	0.95395200	C	6.80639800	1.60939900	0.95402600
C	9.61024300	-0.02434200	-0.04136000	C	9.60982200	-0.02548100	-0.04224800
C	9.11370900	0.54602200	-1.28538900	C	9.10965000	0.54232300	-1.28439600
C	8.17025500	1.65582400	-0.90653600	C	8.17203800	1.65651400	-0.90645900
C	9.12023000	-1.38623300	0.11429200	C	9.11686600	-1.38546600	0.11186300
C	8.26240700	-1.59477900	-0.96999200	C	8.26108300	-1.59574600	-0.97082300
C	8.25819600	-0.42346100	-1.81759800	C	8.25698200	-0.42505000	-1.81896400
C	6.87070400	-1.78477600	-0.50104900	C	6.87091900	-1.78145600	-0.49872700
C	6.00349900	-0.89551100	-1.22489600	C	6.00500400	-0.89019800	-1.21777000
C	6.86375400	0.07422800	-1.84728800	C	6.86434500	0.07480800	-1.84356300
C	6.82876700	1.41163900	-1.31889100	C	6.83209500	1.41154400	-1.31692500
C	5.90151500	1.65588400	-0.14531800	C	5.90626700	1.65429600	-0.14124800
Si	5.14268300	0.04177400	0.07515100	Si	5.13484700	0.04471700	0.07762900
**$C_{19}Si$-CO_2**							
$F = 0.000$				$F = 0.000$			
$E_{ads}(BSSE) = -0.06827564$				$E_{ads}(BSSE) = -0.07742641$			
C	-0.44956200	-0.05900700	-0.12760900	C	-0.26804500	0.10709200	-0.28998600
O	-0.69772900	-0.29614200	0.98521300	O	-0.72140800	-0.26570200	0.70588900
O	-0.23344200	0.17451900	-1.24866500	O	0.15830400	0.48185700	-1.29904000
C	6.95651900	-1.39165200	1.20360200	C	7.08254700	-1.35536100	1.17685600
C	5.52910600	-1.60788700	0.97902400	C	5.65973000	-1.65581400	1.04510600
C	7.08667300	-0.11025000	1.78487900	C	7.17426400	-0.06525500	1.74238100
C	5.73615500	0.43500500	1.90584000	C	5.80598000	0.40172800	1.94720700
C	4.75618300	-0.52928900	1.51216500	C	4.86247400	-0.61993900	1.62105500
C	7.84214900	0.69165400	0.86452200	C	7.82108900	0.77204600	0.77491700
C	6.99122600	1.77351300	0.45643700	C	6.88483500	1.79718600	0.41622100
C	5.67653800	1.61417000	1.07390900	C	5.62487100	1.56727700	1.11727600
C	8.32581500	-0.08312500	-0.26933200	C	8.27189400	0.01677100	-0.38435100
C	7.54225300	0.48848700	-1.35497200	C	7.39241900	0.53430600	-1.42203700
C	6.80285500	1.64594700	-0.93827400	C	6.61619600	1.64827200	-0.96124400
C	7.63523200	-1.34799300	-0.06046100	C	7.67486700	-1.28516600	-0.12697700
C	6.64947800	-1.59964100	-1.07311900	C	6.64351700	-1.59856800	-1.07253100
C	6.59135100	-0.44600400	-1.88696900	C	6.46652700	-0.45779200	-1.88468700
C	5.33676700	-1.73861600	-0.44664800	C	5.38498800	-1.80811300	-0.36252400
C	4.42566000	-0.75366400	-0.94110000	C	4.39019800	-0.88153200	-0.80064200
C	5.24358400	0.10120100	-1.74461100	C	5.10254400	0.01168200	-1.65813800
C	5.37630000	1.41081300	-1.15002200	C	5.19598100	1.32945500	-1.08041300
C	4.65373100	1.49959200	0.08104700	C	4.55108300	1.38501600	0.19297500
Si	3.25102100	0.12812000	0.39547000	Si	3.25300600	-0.05969500	0.60301100
**$C_{19}Si$-N_2**							
$F = 0.000$				$F = 0.000$			
$E_{ads}(BSSE) = -0.01722895$				$E_{ads}(BSSE) = -0.04477245$			
N	-2.66042400	-0.12112300	-0.02744200	N	-1.31609500	0.56939400	-1.66307200
N	-2.14664100	-0.25597700	-0.99254600	N	-0.23966900	0.72589600	-1.56415600
C	5.53073000	-1.36769300	1.24568000	C	5.74786800	-1.18227700	1.30374800
C	4.09734300	-1.57680900	1.05314900	C	4.33401400	-1.46336600	1.53817700
C	5.68206700	-0.07708100	1.80067100	C	5.93814000	0.19077800	1.56973700

C	4.33835200	0.48106700	1.93782900	C	4.63730400	0.72654000	1.96223900
C	3.34315600	-0.48192200	1.58018500	C	3.66086400	-0.31237300	2.05081100
C	6.42540400	0.70185800	0.85109100	C	6.35139800	0.80283600	0.34089700
C	5.57471300	1.78272700	0.43998300	C	5.34476700	1.76237500	-0.00784900
C	4.27128100	1.64500600	1.08598500	C	4.26593400	1.70954600	0.97492200
C	6.88079900	-0.09735900	-0.27775000	C	6.55701300	-0.17846700	-0.71437700
C	6.07996500	0.46064600	-1.35813400	C	5.46879700	0.14834600	-1.62411100
C	5.35780100	1.63132100	-0.94812300	C	4.79136400	1.35203200	-1.23982000
C	6.18465500	-1.35272900	-0.03199400	C	6.04810400	-1.38695800	-0.08325700
C	5.17699400	-1.61481300	-1.02003200	C	4.84410600	-1.85219100	-0.70766000
C	5.11145100	-0.47579400	-1.85367500	C	4.48101200	-0.88953800	-1.67391600
C	3.87583900	-1.73200000	-0.36550900	C	3.76858400	-1.88183100	0.27963700
C	2.96229100	-0.74824200	-0.85875100	C	2.68904100	-1.03210800	-0.11287400
C	3.77089700	0.08462400	-1.69453100	C	3.18925600	-0.34676000	-1.26185100
C	3.92542600	1.40358300	-1.12726900	C	3.38348800	1.05537400	-0.99033300
C	3.22787600	1.51957900	0.11602500	C	3.02269400	1.37758100	0.35379000
Si	1.81656300	0.16712900	0.48402400	Si	1.85855400	0.08117100	1.30818500
**C_{19}Si-CO**							
$F = 0.001$				$F = 0.001$			
$E_{ads}(BSSE) = -0.74916933$				$E_{ads}(BSSE) = -0.78821735$			
O	1.99978300	-0.01200700	-0.00168200	O	2.00507900	-0.01622700	0.01401400
C	3.12640300	-0.00644800	0.00977500	C	3.12040700	-0.00712700	0.02868000
C	8.18551700	-1.37801600	1.28937700	C	8.18555300	-1.37796100	1.29035100
C	6.84233700	-1.69669200	0.93882600	C	6.84434900	-1.69531900	0.93850200
C	8.18666000	-0.09188400	1.86270000	C	8.18727300	-0.09223700	1.86004800
C	6.81531800	0.40070300	1.83327400	C	6.81642400	0.40056400	1.83261800
C	5.91679600	-0.65941800	1.54123300	C	5.91936800	-0.65558200	1.53917900
C	8.99970500	0.72205600	0.99011600	C	8.99929700	0.72197000	0.99030500
C	8.17919200	1.79968800	0.48930800	C	8.18023800	1.79709100	0.48809100
C	6.81044300	1.61202500	0.95347400	C	6.81183100	1.61169500	0.95292900
C	9.61209200	-0.03030800	-0.04917600	C	9.61099800	-0.03032900	-0.04887700
C	9.11234600	0.53936300	-1.29160900	C	9.10854000	0.53679800	-1.29015700
C	8.17371300	1.65290600	-0.91112000	C	8.17438100	1.65352000	-0.91075600
C	9.12022900	-1.39116600	0.10979300	C	9.11572100	-1.38892800	0.10793500
C	8.25638500	-1.59804700	-0.97128800	C	8.25565500	-1.59886100	-0.97262600
C	8.25204300	-0.42901100	-1.82046500	C	8.25155200	-0.42988300	-1.82150600
C	6.86582500	-1.78403700	-0.49663300	C	6.86653000	-1.78101700	-0.49566700
C	5.99899400	-0.89117800	-1.21569900	C	6.00071000	-0.88754200	-1.21121400
C	6.85888800	0.07305400	-1.84523700	C	6.85993700	0.07344300	-1.84235400
C	6.83032100	1.41092800	-1.31767200	C	6.83286200	1.41050300	-1.31680800
C	5.90760900	1.65800800	-0.14157100	C	5.91070800	1.65522600	-0.13886500
Si	5.13686400	0.04838600	0.08314200	Si	5.13005000	0.04910800	0.08358400
**C_{19}Si-CO_2**							
$F = 0.001$				$F = 0.001$			
$E_{ads}(BSSE) = -0.08110533$				$E_{ads}(BSSE) = -0.08655367$			
C	0.29581300	0.39203400	-0.74958400	C	-4.78429600	-0.42291500	-0.00002200
O	-0.66453900	0.12557900	-0.15065000	O	-5.66717600	0.32066100	-0.00040100
O	1.25049100	0.66396800	-1.36292000	O	-3.90664200	-1.18018700	0.00035200
C	7.32425600	-1.27519600	1.03703200	C	2.43400700	1.19711100	0.70601000
C	5.93969600	-1.72597800	1.16341700	C	1.09506300	1.60211300	1.12571600
C	7.37972300	0.02537000	1.58855500	C	2.43388100	1.19714000	-0.70624400
C	6.02786500	0.34651800	2.04196300	C	1.09486200	1.60215800	-1.12569600

C	5.15270100	-0.77336400	1.88341600	C	0.29187500	1.96058600	0.00008900
C	7.74682900	0.91511500	0.52412000	C	2.67324900	-0.15250200	-1.12569600
C	6.65723200	1.83381000	0.35137000	C	1.51508100	-0.57552300	-1.85771500
C	5.57572900	1.47809800	1.26723000	C	0.52002900	0.49295000	-1.84565400
C	8.06223500	0.20063900	-0.70515400	C	2.95690500	-1.03119100	-0.00020900
C	6.95487700	0.61010100	-1.55953100	C	1.77614900	-1.88460700	-0.00012100
C	6.15982900	1.64165000	-0.95726300	C	0.95286400	-1.66076300	-1.15175300
C	7.65888400	-1.15419200	-0.35331900	C	2.67345000	-0.15254700	1.12536500
C	6.51189600	-1.58768200	-1.09938900	C	1.51541100	-0.57559700	1.85757300
C	6.06977900	-0.47948700	-1.85663000	C	0.95306900	-1.66080900	1.15166700
C	5.43120200	-1.92110800	-0.17405300	C	0.52035700	0.49287600	1.84573200
C	4.27880200	-1.10955100	-0.41609700	C	-0.69603600	0.05388600	1.23857500
C	4.72580300	-0.15472200	-1.38219900	C	-0.37734300	-1.23830900	0.72025000
C	4.78199800	1.17257100	-0.81942100	C	-0.37747100	-1.23828000	-0.72008100
C	4.37578500	1.17253800	0.55146200	C	-0.69625600	0.05393600	-1.23829700
Si	3.33097900	-0.39695900	1.17797100	Si	-1.58989700	1.32271000	0.00024500

$C_{19}Si$-N_2

$F = 0.001$, $E_{ads}(BSSE) = -0.04514641$				$F = 0.001$, $E_{ads}(BSSE) = -0.04598611$			
N	-1.31510200	1.05683100	-1.32379700	N	-5.37064000	-0.63226000	-0.11732600
N	-0.29099500	0.65716800	-1.39524700	N	-4.31001800	-0.69445300	0.13647900
C	5.88944400	-1.14395200	1.17613500	C	2.32574400	0.97064100	0.56838900
C	4.52012600	-1.60758400	1.39184400	C	1.08579800	1.60432000	1.00930200
C	5.92197600	0.21696400	1.55676500	C	2.25937900	0.88581100	-0.83949100
C	4.57203000	0.56154900	1.99842400	C	0.98007000	1.46919500	-1.23526900
C	3.72298200	-0.58939600	2.00282200	C	0.29412100	2.01446900	-0.10694200
C	6.24347800	0.97265100	0.37972300	C	2.26936400	-0.50728700	-1.17720900
C	5.12654100	1.83563900	0.11853400	C	1.02696300	-0.78719400	-1.83603100
C	4.07390600	1.57409400	1.09781800	C	0.20901800	0.42253400	-1.85810200
C	6.55184200	0.11495200	-0.75644200	C	2.46717100	-1.35025800	-0.00670600
C	5.41635000	0.38601900	-1.62896000	C	1.17098700	-2.00737200	0.09386100
C	4.60722700	1.46680400	-1.14289600	C	0.33883200	-1.72716300	-1.03907300
C	6.19186400	-1.19318300	-0.22595700	C	2.37494000	-0.37210800	1.06789600
C	5.04130600	-1.74518700	-0.88290400	C	1.20131900	-0.56413600	1.86875600
C	4.55432300	-0.75309100	-1.76372000	C	0.44682600	-1.58903200	1.25794800
C	3.98909000	-1.98311800	0.10304200	C	0.38222900	0.64423900	1.82258200
C	2.81057500	-1.23578100	-0.21126400	C	-0.91425400	0.36520800	1.29027300
C	3.21268700	-0.40241400	-1.30176600	C	-0.82104000	-0.99016500	0.84867900
C	3.24567900	0.98675700	-0.91324800	C	-0.88871800	-1.07664200	-0.58733300
C	2.86742300	1.15057900	0.45624800	C	-1.03041900	0.21657600	-1.17718100
Si	1.87378800	-0.35030200	1.30371400	Si	-1.66467000	1.68265500	0.00570200

$C_{19}Si$-CO

$F = 0.005$, $E_{ads}(BSSE) = -0.95684190$				$F = 0.005$, $E_{ads}(BSSE) = -1.00925177$			
O	1.97416600	0.03725900	-0.02547300	O	1.97940500	0.02780700	-0.01109300
C	3.09787900	0.04225200	0.00046800	C	3.09172700	0.04023200	0.01880800
C	8.17257100	-1.38106500	1.28549900	C	8.17245300	-1.38135500	1.28655100
C	6.82584300	-1.68061900	0.93336700	C	6.82726900	-1.67842200	0.93335000
C	8.18676800	-0.09636500	1.86358100	C	8.18744000	-0.09685100	1.86046700
C	6.81631600	0.40944900	1.83425700	C	6.81715000	0.40999300	1.83416000
C	5.91159600	-0.63408000	1.53486300	C	5.91372800	-0.62876500	1.53313700
C	9.00422600	0.71080000	0.99299600	C	9.00433200	0.71064500	0.99351000

C	8.20134500	1.79857600	0.49398100	C	8.20348100	1.79560400	0.49307100
C	6.82538400	1.62561400	0.95533300	C	6.82740100	1.62640700	0.95536000
C	9.61391500	-0.04749200	-0.05082300	C	9.61316800	-0.04842700	-0.05131200
C	9.12214900	0.53048300	-1.29244700	C	9.11868200	0.52719900	-1.29182200
C	8.19602700	1.65304300	-0.90715700	C	8.19798800	1.65364500	-0.90678500
C	9.10561100	-1.40149900	0.10432000	C	9.10036800	-1.39948600	0.10210600
C	8.23615900	-1.59659000	-0.97895800	C	8.23425000	-1.59773200	-0.98078200
C	8.24543700	-0.42799300	-1.82206200	C	8.24418900	-0.42940400	-1.82374800
C	6.84397400	-1.76701800	-0.50166100	C	6.84370200	-1.76307700	-0.50036800
C	5.98854000	-0.85768400	-1.20999900	C	5.98975200	-0.85279000	-1.20510300
C	6.85830700	0.09230700	-1.84483900	C	6.85923200	0.09338400	-1.84155800
C	6.84945400	1.42717700	-1.31243200	C	6.85307500	1.42734600	-1.31104300
C	5.92946100	1.67716200	-0.13564200	C	5.93415700	1.67524300	-0.13242400
Si	5.11337300	0.08274800	0.08423300	Si	5.10555300	0.08526700	0.08546400
**$C_{19}Si$-CO_2**							
$F = 0.005$<br>$E_{ads}(BSSE) = -0.08494219$				$F = 0.005$<br>$E_{ads}(BSSE) = -0.09138179$			
C	0.32708100	0.45075200	-0.85467000	C	0.30080200	0.47149500	-0.85094400
O	-0.74916700	0.24831900	-0.47170700	O	-0.78330300	0.30851100	-0.49972000
O	1.41181700	0.65407600	-1.24127000	O	1.39542200	0.63560300	-1.20517100
C	7.37530300	-1.28206700	0.97269100	C	7.38519000	-1.27325800	0.97407900
C	6.00364900	-1.76369800	1.13139800	C	6.01746400	-1.76407600	1.13165800
C	7.42338000	0.00544100	1.55977300	C	7.42417500	0.01382300	1.55924900
C	6.07987500	0.28503800	2.06534900	C	6.07893200	0.28489000	2.06238800
C	5.21973600	-0.84612100	1.89973200	C	5.22728700	-0.85116400	1.89642000
C	7.73572600	0.93129300	0.50983800	C	7.73215800	0.94148800	0.51166900
C	6.62262600	1.83086800	0.39823500	C	6.61286600	1.83055400	0.39572800
C	5.57908700	1.42578800	1.33824900	C	5.57212800	1.41945500	1.33492100
C	8.02770000	0.25802500	-0.75107600	C	8.02562600	0.26701400	-0.74772300
C	6.87911400	0.66828600	-1.55705800	C	6.87785000	0.66966800	-1.55837000
C	6.08527900	1.66318700	-0.89811600	C	6.07828600	1.65785200	-0.89957100
C	7.65949700	-1.11535600	-0.42318200	C	7.67087800	-1.10989000	-0.42023200
C	6.49688100	-1.55072500	-1.14332200	C	6.51189500	-1.54988800	-1.14008500
C	6.00745400	-0.43044600	-1.85266000	C	6.01600600	-0.43533700	-1.85128500
C	5.45422600	-1.93076000	-0.19183600	C	5.47178500	-1.93610000	-0.18984600
C	4.27769300	-1.13545200	-0.36818100	C	4.29174200	-1.14895100	-0.36848100
C	4.67232000	-0.14729400	-1.32438300	C	4.67961700	-0.16066800	-1.32501000
C	4.72087000	1.16161300	-0.72788000	C	4.71857500	1.14665200	-0.73063000
C	4.36117700	1.11101600	0.65562700	C	4.35910600	1.09389600	0.65143700
Si	3.35653700	-0.49203400	1.27477000	Si	3.36337400	-0.51181900	1.26983900
**$C_{19}Si$-N_2**							
$F = 0.005$<br>$E_{ads}(BSSE) = -0.05377844$				$F = 0.005$<br>$E_{ads}(BSSE) = -0.05636742$			
N	-1.32195000	0.90194600	-1.47271000	N	-1.32775300	0.87494400	-1.48440400
N	-0.24969700	0.68184500	-1.34816100	N	-0.26102300	0.69182300	-1.33755900
C	5.88013300	-1.17120100	1.15892300	C	5.87753900	-1.17285600	1.15726300
C	4.50333900	-1.61059600	1.38401100	C	4.50047800	-1.60921400	1.38135500
C	5.94130000	0.18757900	1.55051900	C	5.94146100	0.18415600	1.54941700
C	4.60081900	0.55256000	2.00702900	C	4.60243600	0.55176600	2.00550600
C	3.72850600	-0.58160900	2.00679000	C	3.72876900	-0.57947400	2.00342600
C	6.26392400	0.94625300	0.37652100	C	6.26668400	0.94435500	0.37902200
C	5.16044200	1.83067900	0.13207200	C	5.16480600	1.82800900	0.13333500

C	4.11217900	1.57910400	1.11955900	C	4.11692200	1.57815200	1.12011300
C	6.54967000	0.09192600	-0.77122000	C	6.54657900	0.08867700	-0.76864500
C	5.40575600	0.39069700	-1.63105800	C	5.40619300	0.39122800	-1.63154500
C	4.62203300	1.47993500	-1.12699600	C	4.62656100	1.47988000	-1.12479100
C	6.16716300	-1.21456800	-0.24570700	C	6.16520600	-1.21867200	-0.24551600
C	5.00025800	-1.73912700	-0.89591600	C	4.99732200	-1.73661200	-0.89598500
C	4.52283400	-0.73066500	-1.76347800	C	4.52282300	-0.72784600	-1.76216500
C	3.95299800	-1.96446500	0.09909600	C	3.95056700	-1.96044100	0.09833500
C	2.78476800	-1.19110400	-0.19406200	C	2.78590100	-1.18392200	-0.19386200
C	3.19076400	-0.35925300	-1.28497100	C	3.19241700	-0.35363000	-1.28383000
C	3.25284100	1.02280200	-0.88693500	C	3.25728100	1.02533600	-0.88559200
C	2.89139800	1.17856000	0.48864400	C	2.89716600	1.17987500	0.48893100
Si	1.86706300	-0.30530100	1.33691800	Si	1.86820500	-0.29953500	1.33605800
**$C_{19}Si$-CO**							
$F = 0.010$<br>$E_{ads}(BSSE) = -1.26088602$				$F = 0.010$<br>$E_{ads}(BSSE) = -1.32802545$			
O	1.94882700	-0.02443100	-0.01647800	O	1.95599700	-0.02593500	0.00455300
C	3.06987600	-0.01053700	0.01228300	C	3.06579600	-0.00796000	0.02853600
C	8.19175900	-1.38011600	1.30854900	C	8.20031700	-1.37791900	1.28911100
C	6.84930400	-1.67015400	0.93821400	C	6.85759600	-1.68726600	0.93584500
C	8.19355300	-0.08778500	1.87785400	C	8.19800000	-0.09123100	1.86040500
C	6.81899100	0.39648800	1.83676100	C	6.81523700	0.39754900	1.83441500
C	5.91834300	-0.66014200	1.55065900	C	5.93264200	-0.64483900	1.52640400
C	9.00590600	0.72430900	0.99745100	C	9.00102900	0.72542200	0.99538700
C	8.20209900	1.79249400	0.48630100	C	8.19148200	1.79727700	0.48933100
C	6.81724400	1.60983800	0.95359200	C	6.81093800	1.61489200	0.95043000
C	9.62699800	-0.02463100	-0.05436000	C	9.62476600	-0.03298600	-0.05197800
C	9.16432800	0.55302700	-1.32861100	C	9.12110600	0.53315200	-1.29224600
C	8.20275900	1.62550100	-0.91187800	C	8.18988200	1.65274900	-0.91094000
C	9.08893600	-1.34990100	0.10571700	C	9.12832100	-1.39068700	0.10399600
C	8.24847100	-1.60132100	-0.99556400	C	8.25677500	-1.59832200	-0.97963600
C	8.25347500	-0.41959900	-1.80615000	C	8.25293100	-0.43683200	-1.82329300
C	6.86024400	-1.77390200	-0.50648600	C	6.86821400	-1.77896900	-0.49553200
C	5.99643700	-0.88098700	-1.20052600	C	6.00546400	-0.87173500	-1.19045100
C	6.85913000	0.08081000	-1.85112200	C	6.86201000	0.07348500	-1.84059500
C	6.84711700	1.40385100	-1.32181300	C	6.84696900	1.40607600	-1.30994600
C	5.93738600	1.61686400	-0.13362000	C	5.92457000	1.64093000	-0.13304500
Si	5.08628500	0.02922400	0.09809200	Si	5.07742800	0.05205000	0.08665200
**$C_{19}Si$-CO_2**							
$F = 0.010$<br>$E_{ads}(BSSE) = -0.10993079$				$F = 0.010$<br>$E_{ads}(BSSE) = -0.12354919$			
C	0.27117300	0.53068500	-0.95472800	C	0.28395900	0.61706600	-0.93352700
O	-0.88470100	0.52655700	-0.91370700	O	-0.85715100	0.73365700	-0.93228800
O	1.44377200	0.53612200	-0.99831000	O	1.44542800	0.50391400	-0.93712200
C	7.45390500	-1.25189700	0.93438500	C	7.48386300	-1.21288500	0.94499900
C	6.10211900	-1.77145700	1.14691700	C	6.14778100	-1.77209700	1.15557200
C	7.48689800	0.04081800	1.51837000	C	7.47363100	0.08364800	1.51862000
C	6.15433800	0.28143300	2.07391500	C	6.13216300	0.28635200	2.06660400
C	5.31829200	-0.87268000	1.93877200	C	5.33390300	-0.89310300	1.93656900
C	7.72905600	0.97175600	0.45562400	C	7.69262000	1.01565000	0.45391100
C	6.58534100	1.83672100	0.38478000	C	6.52141800	1.83924500	0.36891700
C	5.59105800	1.40158100	1.36490100	C	5.53840900	1.38105400	1.34815700

C	7.99665500	0.30411200	-0.81758500	C	7.98140000	0.34451800	-0.81301700
C	6.79865600	0.67832200	-1.57953300	C	6.77801000	0.67724000	-1.58655500
C	6.00285600	1.64717300	-0.88922900	C	5.95177200	1.62411800	-0.90486400
C	7.67711300	-1.08038200	-0.47093900	C	7.70854900	-1.04817400	-0.45937300
C	6.49959400	-1.55022000	-1.14435100	C	6.54759800	-1.55483300	-1.13275000
C	5.94996400	-0.44600500	-1.83418400	C	5.96811000	-0.47479700	-1.83334400
C	5.50581000	-1.95613100	-0.15089300	C	5.56427300	-1.98389700	-0.14094900
C	4.29853600	-1.19501400	-0.27727200	C	4.33611800	-1.26004800	-0.27711700
C	4.62610500	-0.20117800	-1.25434700	C	4.63560800	-0.26611400	-1.26084600
C	4.65925600	1.10615300	-0.66415800	C	4.62575500	1.04274700	-0.68175100
C	4.35561400	1.04371300	0.73338400	C	4.31937100	0.97958900	0.71414300
Si	3.40645100	-0.58043300	1.39850900	Si	3.41527400	-0.66310000	1.38633500
**C_{19}Si-N_2**							
$F = 0.010$				$F = 0.010$			
$E_{ads}(BSSE) = -0.07166555$				$E_{ads}(BSSE) = -0.07659200$			
N	-1.26616900	0.80685300	-1.54125200	N	-1.26724600	0.80628400	-1.52147400
N	-0.20408800	0.65325200	-1.29151400	N	-0.21188800	0.65604400	-1.28266800
C	5.87099400	-1.18491500	1.14733100	C	5.86792600	-1.18344900	1.14908200
C	4.48883800	-1.60970000	1.37624500	C	4.48560300	-1.60788500	1.37378700
C	5.94846900	0.17372300	1.54606300	C	5.94400900	0.17427200	1.54699000
C	4.61179600	0.54954700	2.00889200	C	4.60650100	0.55021500	2.00535800
C	3.72441200	-0.57402800	2.00258300	C	3.72027400	-0.57245800	1.99629300
C	6.27207400	0.93394900	0.37427300	C	6.27229400	0.93592600	0.37927000
C	5.17606000	1.82984700	0.13798900	C	5.17559200	1.82736800	0.13722200
C	4.12914300	1.58240700	1.12893800	C	4.12710800	1.58052200	1.12500800
C	6.54947100	0.08196500	-0.78157400	C	6.54791400	0.08117700	-0.77499700
C	5.39750000	0.39694400	-1.63534900	C	5.40108000	0.39665700	-1.63624300
C	4.62793800	1.48854800	-1.12001000	C	4.63198700	1.48627200	-1.12105600
C	6.15028000	-1.22450400	-0.25784000	C	6.15208900	-1.22723300	-0.25338300
C	4.97441000	-1.73196500	-0.90523200	C	4.97745900	-1.73033400	-0.90347800
C	4.50342500	-0.71365800	-1.76484800	C	4.50958200	-0.71392200	-1.76439100
C	3.92878300	-1.94962500	0.09438100	C	3.93030200	-1.94772000	0.09293800
C	2.76599100	-1.16061500	-0.18522700	C	2.77008900	-1.15810900	-0.18952300
C	3.17510100	-0.33100100	-1.27800200	C	3.18071400	-0.33069800	-1.28171400
C	3.25299200	1.04447600	-0.87518000	C	3.25719600	1.04181900	-0.88025900
C	2.89994600	1.19256700	0.50442100	C	2.90140900	1.18907000	0.49736100
Si	1.84917300	-0.27807000	1.35378100	Si	1.84654500	-0.27782100	1.34474600
**C_{19}Si-CO**							
$F = 0.015$				$F = 0.015$			
$E_{ads}(BSSE) = -1.60888130$				$E_{ads}(BSSE) = -1.69386335$			
O	1.91954500	-0.01286000	0.00018700	O	1.92700900	-0.02083000	0.01198100
C	3.03892700	-0.00811300	0.02064100	C	3.03498500	-0.00583200	0.03150900
C	8.19503700	-1.37817300	1.32130700	C	8.20964800	-1.37828400	1.28856400
C	6.84495900	-1.64676800	0.93330700	C	6.86551100	-1.68339400	0.93183200
C	8.19605300	-0.08275000	1.89186100	C	8.20193300	-0.09072500	1.85864400
C	6.82490500	0.39398500	1.83331000	C	6.81212400	0.39537100	1.83313000
C	5.91599800	-0.67108000	1.54832400	C	5.93809100	-0.64139400	1.51459000
C	9.01740200	0.73367900	1.00970500	C	9.00311700	0.72951000	1.00052600
C	8.21823300	1.79172200	0.48731600	C	8.19481500	1.79636200	0.48959700
C	6.82533700	1.60084500	0.95432500	C	6.80757500	1.61446200	0.94778500
C	9.64121500	-0.01069500	-0.04652800	C	9.63446900	-0.03216500	-0.05092500
C	9.20335100	0.57381000	-1.34913000	C	9.12890900	0.53314500	-1.29107400

C	8.22717400	1.60400200	-0.90927800	C	8.19825800	1.65406200	-0.91137400
C	9.05788000	-1.30813100	0.11235300	C	9.13946600	-1.39234300	0.10429300
C	8.24046500	-1.59401200	-1.00789200	C	8.26071900	-1.59656800	-0.98148800
C	8.25274400	-0.40926800	-1.79399400	C	8.25642300	-0.44013600	-1.82318500
C	6.84883700	-1.77162000	-0.52237300	C	6.87162900	-1.78035200	-0.49711800
C	5.98790200	-0.89156800	-1.20973200	C	6.01053200	-0.86548800	-1.18168700
C	6.86054300	0.07822900	-1.86772600	C	6.86493800	0.07166000	-1.84192900
C	6.85729300	1.38530400	-1.32343800	C	6.85425400	1.40078200	-1.30627300
C	5.95950800	1.57230800	-0.13712400	C	5.92879900	1.63050600	-0.13347500
Si	5.05415900	0.00005400	0.09344200	Si	5.04426400	0.05055200	0.08348300

$C_{19}Si$-CO_2

$F = 0.015$<br>$E_{ads}(BSSE) = -0.16062570$				$F = 0.015$<br>$E_{ads}(BSSE) = -0.19373070$			
C	0.29735800	0.52793600	-0.91562600	C	-4.83873800	-0.41781100	0.00001900
O	-0.85237700	0.58457000	-0.98263000	O	-5.98104900	-0.38178600	-0.00014700
O	1.47263100	0.47367500	-0.85278800	O	-3.66661500	-0.45570500	0.00018600
C	7.44603000	-1.23526300	0.96595600	C	2.60731400	0.93744500	0.71089600
C	6.08790300	-1.73200400	1.20336900	C	1.33686100	1.53737500	1.12564600
C	7.49373900	0.08119700	1.50145100	C	2.60712900	0.93722100	-0.71160500
C	6.16308400	0.35399500	2.05098300	C	1.33657600	1.53702900	-1.12622800
C	5.31186500	-0.79458100	1.95900500	C	0.58494000	2.00027200	-0.00026800
C	7.73809500	0.96798300	0.40305500	C	2.63587600	-0.43164600	-1.12937400
C	6.60051300	1.83880300	0.30284500	C	1.42097100	-0.67737900	-1.85422500
C	5.60608300	1.44814500	1.30224400	C	0.59829600	0.52977900	-1.83621500
C	8.00034400	0.25018400	-0.84844100	C	2.79388300	-1.35075700	-0.00002600
C	6.79415700	0.60657100	-1.61951900	C	1.47760400	-2.02002800	0.00024000
C	6.01061600	1.60400000	-0.96045500	C	0.70262400	-1.66260400	-1.14361100
C	7.66338900	-1.11926000	-0.44490700	C	2.63615600	-0.43130000	1.12907200
C	6.47718300	-1.60109200	-1.09469400	C	1.42143100	-0.67681400	1.85431200
C	5.93470400	-0.51785100	-1.82230400	C	0.70290600	-1.66225800	1.14416900
C	5.48362100	-1.95792200	-0.08146300	C	0.59875800	0.53034100	1.83613100
C	4.28047100	-1.18999700	-0.22720900	C	-0.67325200	0.29204400	1.21880100
C	4.61266800	-0.23952100	-1.24698200	C	-0.55655900	-1.04227900	0.71338200
C	4.66010400	1.08502200	-0.70909400	C	-0.55673700	-1.04249800	-0.71270400
C	4.36239000	1.07373700	0.69242200	C	-0.67356400	0.29166700	-1.21851600
Si	3.38329300	-0.50857900	1.42510900	Si	-1.41382400	1.68808100	0.00002100

$C_{19}Si$-N_2

$F = 0.015$<br>$E_{ads}(BSSE) = -0.09723814$				$F = 0.015$<br>$E_{ads}(BSSE) = -0.11392979$			
N	-1.26164500	0.75148800	-1.46851600	N	-5.31090100	-0.74408500	0.00023300
N	-0.19483500	0.62199700	-1.22398300	N	-4.22618000	-0.61162400	-0.00024700
C	5.85253300	-1.19301800	1.15460500	C	2.27950300	0.97444900	0.71209100
C	4.46224100	-1.60549000	1.36629200	C	0.99749500	1.54849700	1.12720400
C	5.93638800	0.16728500	1.55653700	C	2.28007100	0.97522900	-0.70973900
C	4.59473000	0.55205900	2.00207700	C	0.99838300	1.54971900	-1.12522800
C	3.69509000	-0.56292200	1.97988400	C	0.23716600	1.99650200	0.00093200
C	6.27868000	0.92529100	0.38962100	C	2.33711100	-0.39263000	-1.12857900
C	5.19108100	1.82830600	0.13998700	C	1.12840500	-0.66217300	-1.85451700
C	4.12992500	1.58606400	1.11791100	C	0.28163000	0.52826900	-1.83667900
C	6.57000500	0.07168500	-0.76703000	C	2.51354500	-1.30937500	0.00002600
C	5.42242800	0.39662600	-1.63453900	C	1.21093300	-2.00472700	-0.00086300
C	4.65524500	1.49013800	-1.12461900	C	0.42961800	-1.66250600	-1.14514800

C	6.14763000	-1.23385400	-0.24611000	C	2.33622600	-0.39385700	1.12950000
C	4.97433800	-1.72914800	-0.90762200	C	1.12695100	-0.66418400	1.85417700
C	4.52171200	-0.70582600	-1.77107200	C	0.42872400	-1.66374400	1.14318900
C	3.91455700	-1.93786200	0.08011400	C	0.28018200	0.52627800	1.83697700
C	2.75939800	-1.13772800	-0.20840400	C	-0.98585400	0.26279500	1.21814700
C	3.18788000	-0.31350800	-1.29981200	C	-0.84213700	-1.06860500	0.71223000
C	3.27142200	1.05695300	-0.89638600	C	-0.84158500	-1.06783600	-0.71453600
C	2.90314200	1.20176400	0.48099300	C	-0.98488900	0.26411600	-1.21910800
Si	1.81459600	-0.25430100	1.31894100	Si	-1.75066400	1.64804700	-0.00002600

C$_{19}$Si-CO

$F = 0.020$<br>$E_{ads}(BSSE) = -1.98886525$				$F = 0.020$<br>$E_{ads}(BSSE) = -2.10539484$			
O	1.88954200	0.00012800	0.02114300	O	1.90102200	-0.01104400	0.02234300
C	3.00771700	-0.00457200	0.02868300	C	3.00793500	-0.00078600	0.03536300
C	8.19856400	-1.37220000	1.32653600	C	8.22171000	-1.37766500	1.28255300
C	6.83366300	-1.62894600	0.93039200	C	6.87463900	-1.68403200	0.92637900
C	8.20196000	-0.07674400	1.90312500	C	8.20635100	-0.09068500	1.85205500
C	6.83746600	0.39577700	1.83171400	C	6.80859700	0.39445000	1.83100000
C	5.91816200	-0.68091400	1.53482200	C	5.94628100	-0.63607100	1.49511900
C	9.03638600	0.74858300	1.02295600	C	9.00633800	0.73558300	1.00593700
C	8.23680900	1.79890100	0.49317200	C	8.19516900	1.79763900	0.49069500
C	6.83940000	1.58790000	0.95628300	C	6.80272300	1.61347900	0.94356100
C	9.65750300	0.00516800	-0.03393400	C	9.64581900	-0.03098600	-0.04797100
C	9.22829800	0.59456400	-1.35402000	C	9.12431300	0.53008700	-1.27813500
C	8.25093200	1.59378000	-0.90349100	C	8.20353100	1.66232100	-0.91174500
C	9.03338200	-1.27578200	0.12173900	C	9.16420600	-1.40606400	0.10654800
C	8.22934200	-1.57939500	-1.01099100	C	8.26644300	-1.59247700	-0.97692300
C	8.24730500	-0.40253400	-1.79178800	C	8.25874200	-0.44704500	-1.82490500
C	6.83214900	-1.77373200	-0.53944600	C	6.87694500	-1.78517800	-0.49707200
C	5.97629200	-0.90933400	-1.22594800	C	6.01505600	-0.86146700	-1.17556100
C	6.86395700	0.07006000	-1.89047700	C	6.86604600	0.06631300	-1.84223600
C	6.86713200	1.36014000	-1.31818000	C	6.85838200	1.39378700	-1.29808400
C	5.98082000	1.52679900	-0.14236600	C	5.92711300	1.62630800	-0.13635200
Si	5.02068600	-0.02874600	0.07893700	Si	5.01010800	0.05243600	0.07483300

C$_{19}$Si-CO$_2$

$F = 0.020$<br>$E_{ads}(BSSE) = -0.23431939$				$E_{ele} = 0.020$<br>$E_{ads}(BSSE) = -0.27245444$			
C	0.32322500	0.49300400	-0.83982400	C	0.33691300	0.49228500	-0.83160700
O	-0.82158300	0.55334000	-0.92393000	O	-0.79530900	0.56174200	-0.93425400
O	1.50299400	0.43517800	-0.76015000	O	1.51025000	0.42470900	-0.73249100
C	7.41995500	-1.24375000	0.98093600	C	7.41748500	-1.24178200	0.97985100
C	6.05023300	-1.72600300	1.19295000	C	6.04740300	-1.72423300	1.19081300
C	7.46952500	0.07549700	1.51841900	C	7.46553800	0.07713800	1.51671300
C	6.12785900	0.35845300	2.04098600	C	6.12284700	0.35902200	2.03773400
C	5.26409500	-0.78029600	1.92956000	C	5.26113100	-0.77959400	1.92606900
C	7.74082000	0.95898500	0.42511200	C	7.74047800	0.96407600	0.42907300
C	6.61066000	1.83732400	0.30200200	C	6.60553800	1.83425200	0.30013300
C	5.59336500	1.45315800	1.28180600	C	5.58947800	1.45034700	1.27924300
C	8.02837400	0.23869000	-0.82561800	C	8.02154800	0.23871700	-0.82204000
C	6.83073200	0.60557000	-1.61949300	C	6.82707900	0.60740900	-1.62406100
C	6.04317600	1.60527800	-0.97230300	C	6.04116100	1.60391900	-0.97410900
C	7.66388900	-1.13027400	-0.42450700	C	7.66490800	-1.13476300	-0.42383100

C	6.48356400	-1.59896000	-1.09544500	C	6.48192900	-1.59584800	-1.09384000
C	5.96517800	-0.51046100	-1.83247900	C	5.96513700	-0.50989300	-1.83272000
C	5.46725300	-1.94446600	-0.09981100	C	5.46696400	-1.94229200	-0.09933200
C	4.27096300	-1.16510600	-0.26438700	C	4.27201900	-1.16084000	-0.26348000
C	4.63142700	-0.22072000	-1.28307900	C	4.63122600	-0.21943000	-1.28480900
C	4.68030500	1.09939200	-0.74656200	C	4.67866400	1.09646400	-0.75037600
C	4.35507800	1.08572800	0.65089300	C	4.35337800	1.07910500	0.64656500
Si	3.32678100	-0.47981300	1.36525200	Si	3.32210000	-0.48076100	1.36108300
**$C_{19}Si-N_2$**							
$F = 0.020$<br>$E_{ads}(BSSE) = -0.13186518$				$F = 0.020$<br>$E_{ads}(BSSE) = -0.14614638$			
N	-1.27155400	0.70522700	-1.37834600	N	-1.20947700	0.71534600	-1.37477400
N	-0.20053500	0.58927700	-1.14355600	N	-0.15333000	0.58537200	-1.12337000
C	5.83284100	-1.20001400	1.16501000	C	5.83016800	-1.19641300	1.16224000
C	4.43393100	-1.60039900	1.35539000	C	4.43188700	-1.59891700	1.35357100
C	5.92124400	0.16233300	1.56974100	C	5.91573500	0.16571300	1.56577400
C	4.57286200	0.55546900	1.99328700	C	4.56666600	0.55608500	1.98972100
C	3.66160000	-0.55112500	1.95335200	C	3.65877300	-0.55162200	1.95092900
C	6.28564200	0.91738200	0.40870900	C	6.28109800	0.92487500	0.40954800
C	5.20644700	1.82627500	0.14172100	C	5.19575100	1.82392800	0.13874400
C	4.12812100	1.58924800	1.10312300	C	4.12017600	1.58571900	1.10093500
C	6.59653000	0.06127500	-0.74769400	C	6.58505900	0.06417300	-0.74738800
C	5.45502200	0.39501200	-1.63258000	C	5.44534000	0.39690300	-1.63870300
C	4.68739300	1.48987800	-1.13084700	C	4.67827800	1.48771300	-1.13284000
C	6.14850300	-1.24333300	-0.22983200	C	6.14694000	-1.24596700	-0.23041800
C	4.97979500	-1.72690000	-0.90849100	C	4.97598900	-1.72392000	-0.90739200
C	4.54819200	-0.69964500	-1.77773800	C	4.54310900	-0.70001300	-1.77738100
C	3.90286200	-1.92628800	0.06396600	C	3.90190800	-1.92534500	0.06569200
C	2.75528700	-1.11620100	-0.23800600	C	2.75413500	-1.11534300	-0.23476500
C	3.20712700	-0.29810200	-1.32712300	C	3.20212400	-0.29971300	-1.32666900
C	3.29423800	1.06754400	-0.92375000	C	3.28645100	1.06198400	-0.92546000
C	2.90546400	1.20979900	0.45006600	C	2.89920800	1.20099800	0.44854700
Si	1.77552900	-0.23071300	1.27246600	Si	1.77055200	-0.23555800	1.27232700
**$C_{19}Si-CO$**							
$F = 0.025$<br>$E_{ads}(BSSE) = -2.40028075$				$F = 0.025$<br>$E_{ads}(BSSE) = -2.57870530$			
O	1.85816900	-0.00376100	0.03338800	O	1.86113700	-0.01874700	0.06004300
C	2.97544400	-0.00582500	0.03322200	C	2.96684000	-0.00883200	0.05139700
C	8.20407300	-1.37330600	1.32777100	C	8.21102200	-1.38099200	1.31912800
C	6.83386500	-1.62468900	0.92907300	C	6.85409900	-1.63787900	0.92246500
C	8.20779500	-0.07616600	1.90537100	C	8.20274900	-0.08436100	1.87999800
C	6.83909300	0.39478400	1.82816800	C	6.81261200	0.38848900	1.82095100
C	5.92277500	-0.67575000	1.52215300	C	5.92300900	-0.65589800	1.51393200
C	9.03887500	0.74625500	1.02363700	C	9.02123800	0.73670900	1.02193000
C	8.24130900	1.79873300	0.49187000	C	8.21879100	1.78062900	0.48393000
C	6.83997800	1.58729400	0.95370000	C	6.81565900	1.60348500	0.94728200
C	9.67552400	0.00323900	-0.02800900	C	9.66935000	-0.01820000	-0.03577300
C	9.24468100	0.59704400	-1.35841800	C	9.22352100	0.56838400	-1.34508400
C	8.25895500	1.59186600	-0.90288600	C	8.24181200	1.61781800	-0.91149900
C	9.03374500	-1.27279500	0.12461800	C	9.07905100	-1.31963200	0.11570600
C	8.23214500	-1.57741100	-1.01143400	C	8.25222200	-1.58890500	-1.00516400
C	8.25384000	-0.40200400	-1.79048200	C	8.26894100	-0.41838400	-1.80024000

C	6.83091700	-1.77198800	-0.54169500	C	6.85521800	-1.77110600	-0.52293800
C	5.97926600	-0.90390900	-1.22108300	C	6.00893400	-0.86683800	-1.18752200
C	6.87098800	0.07009700	-1.89119700	C	6.88098900	0.07760300	-1.86728400
C	6.87451100	1.35810400	-1.31824000	C	6.88080700	1.38233300	-1.32119100
C	5.98541900	1.51652200	-0.14376100	C	5.96859500	1.55378800	-0.14166200
Si	4.98610100	-0.02743500	0.07309700	Si	4.97087100	0.00943600	0.07900000
**$C_{19}Si$-CO_2**							
$F = 0.025$				$F = 0.025$			
$E_{ads}(BSSE) = -0.33903162$				$E_{ads}(BSSE) = -0.40174203$			
C	0.31320300	0.45154600	-0.73552100	C	0.30768200	0.43695200	-0.70366100
O	-0.82939200	0.50319000	-0.80162800	O	-0.82237100	0.49545200	-0.78429400
O	1.50139000	0.40247400	-0.67388700	O	1.49178700	0.38007900	-0.62617300
C	7.38570500	-1.25707400	1.00523500	C	7.37581900	-1.25673900	1.01137100
C	6.00062500	-1.71968700	1.17665200	C	5.98717900	-1.71439400	1.17441700
C	7.43587700	0.06409200	1.54700600	C	7.42591000	0.06483100	1.55050900
C	6.07849200	0.36082800	2.02763300	C	6.06530000	0.36615400	2.02117100
C	5.20034200	-0.76472400	1.88708600	C	5.18643200	-0.75627100	1.87668400
C	7.74911600	0.94424400	0.46400500	C	7.75471800	0.94714000	0.47642200
C	6.63033800	1.83286000	0.30668600	C	6.63311700	1.82803100	0.30245200
C	5.57822700	1.45716400	1.25400600	C	5.57495100	1.45799600	1.24267100
C	8.07547000	0.22180000	-0.78391600	C	8.07910900	0.21436000	-0.77058500
C	6.89566800	0.60451800	-1.61336900	C	6.90929400	0.60306900	-1.61923300
C	6.09995100	1.60791400	-0.98491000	C	6.11469800	1.60593200	-0.99297600
C	7.67185900	-1.14613300	-0.39084700	C	7.67601900	-1.15865900	-0.38053500
C	6.50280600	-1.59546600	-1.09514000	C	6.50502400	-1.59320200	-1.08969400
C	6.02190200	-0.49962200	-1.84654500	C	6.03598900	-0.49973600	-1.84930800
C	5.45192600	-1.92614300	-0.12926400	C	5.44839900	-1.91887200	-0.13114500
C	4.26685400	-1.13080300	-0.32565400	C	4.26881500	-1.11547700	-0.33601700
C	4.67223700	-0.19407100	-1.33880500	C	4.68376200	-0.18672700	-1.35406400
C	4.72108800	1.12041400	-0.80165300	C	4.73267400	1.12323900	-0.82152700
C	4.35074700	1.10238900	0.58755500	C	4.35244200	1.10188500	0.56580500
Si	3.25343100	-0.43996100	1.26560400	Si	3.24111400	-0.42529600	1.23803600
**$C_{19}Si$-N_2**							
$F = 0.025$				$F = 0.025$			
$E_{ads}(BSSE) = -0.17736559$				$E_{ads}(BSSE) = -0.20140237$			
N	-1.33534300	0.66360200	-1.26486100	N	-1.29172000	0.66420600	-1.23049000
N	-0.25854600	0.55945800	-1.04850900	N	-0.22750900	0.54873500	-1.00363100
C	5.81248000	-1.20679300	1.18059600	C	5.80378000	-1.20446500	1.18191100
C	4.40407900	-1.59420200	1.34297900	C	4.39421500	-1.59196000	1.33867600
C	5.90395000	0.15796300	1.58773100	C	5.89234900	0.16007900	1.58782900
C	4.54684800	0.56000700	1.98188700	C	4.53304400	0.56175300	1.97606100
C	3.62402200	-0.53771600	1.92063500	C	3.61227900	-0.53568700	1.91087100
C	6.29681400	0.90886600	0.43464400	C	6.29568700	0.91614700	0.44407800
C	5.22744400	1.82302900	0.14416300	C	5.22106900	1.81891900	0.14215800
C	4.12693600	1.59235200	1.08309200	C	4.11802700	1.58939000	1.07597800
C	6.63499500	0.04887000	-0.71964200	C	6.63066700	0.04861000	-0.71065400
C	5.50308000	0.39107200	-1.62777100	C	5.50577200	0.39223400	-1.63346100
C	4.73220500	1.48751100	-1.13862500	C	4.73469800	1.48525500	-1.14303600
C	6.15611600	-1.25438900	-0.20591400	C	6.15685800	-1.26187100	-0.19965000
C	4.99508100	-1.72570500	-0.90687300	C	4.99489800	-1.72216600	-0.90455000
C	4.59001200	-0.69538600	-1.78484900	C	4.59588400	-0.69501600	-1.78680000
C	3.89601700	-1.91432400	0.04447400	C	3.89364100	-1.91081500	0.04160200

C	2.75751300	-1.09433800	-0.27774300	C	2.75808600	-1.08738600	-0.28542000
C	3.23967800	-0.28384600	-1.36275300	C	3.24398600	-0.28178100	-1.37307500
C	3.32882600	1.07707100	-0.96001700	C	3.33052100	1.07440600	-0.97270200
C	2.91128600	1.21780100	0.40787900	C	2.90687200	1.21074900	0.39408000
Si	1.73304800	-0.20490300	1.20834800	Si	1.72343800	-0.20333600	1.18909600

Table S3. Frontier orbital energies (in eV) for C_{20} and $C_{19}Si$ fullerenes. Percentual increase of the HOMO-LUMO gap, ΔE_{HL} is also shown.

B3LYP/6-31G(d)				
	E_H	E_L	E_{HL}	ΔE_{HL}
C_{20}	-5.061	-3.111	1.949	
$C_{19}Si$	-5.610	-3.295	2.316	18.81%
B3LYP/6-311+G(d,p)				
	E_H	E_L	E_{HL}	ΔE_{HL}
C_{20}	-5.537	-3,623	1.914	
$C_{19}Si$	-5.924	-3.050	2.219	15.92%
B3LYP/def2TVZP				
	E_H	E_L	E_{HL}	ΔE_{HL}
C_{20}	-5.443	-3.524	1.919	
$C_{19}Si$	-5.821	-3.615	2.206	14.96%
ωB97XD/6-31G(d)				
	E_H	E_L	E_{HL}	ΔE_{HL}
C_{20}	-7.017	-1.560	5.458	
$C_{19}Si$	-7.537	-1.731	5.805	6.37%
ωB97XD /6-311+G(d,p)				
	E_H	E_L	E_{HL}	ΔE_{HL}
C_{20}	-7.355	-1.960	5.395	
$C_{19}Si$	-7.765	-2.044	5.722	6.06%
ωB97XD /def2TVZP				
	E_H	E_L	E_{HL}	ΔE_{HL}
C_{20}	-7.246	-1.817	5.429	
$C_{19}Si$	-7.655	-1.927	5.727	5.49%
M06L/6-31G(d)				
	E_H	E_L	E_{HL}	ΔE_{HL}
C_{20}	-4.464	-3.607	0.857	
$C_{19}Si$	-5.059	-3.730	1.329	55.10%
M06L/6-311+G(d,p)				
	E_H	E_L	E_{HL}	ΔE_{HL}
C_{20}	-4.710	-3.847	0.862	
$C_{19}Si$	-5.198	-3.901	1.297	50.36%
M06L/def2TVZP				
	E_H	E_L	E_{HL}	ΔE_{HL}
C_{20}	-4,542	-3,652	0,890	
$C_{19}Si$	-5,049	-3,734	1,315	47.80%
M062X/6-31G(d)				

	E_H	E_L	E_{HL}	ΔE_{HL}
C_{20}	-6.310	-2.427	3.883	
$C_{19}Si$	-6.843	-2.589	4.254	9.57%
M062X/6-311+G(d,p)				
	E_H	E_L	E_{HL}	ΔE_{HL}
C_{20}	-6.310	-2.427	3.883	
$C_{19}Si$	-7.104	-2.923	4.181	7.68%
M062X/def2TVZP				
	E_H	E_L	E_{HL}	ΔE_{HL}
C_{20}	-6.540	-2.689	3.851	
$C_{19}Si$	-6.968	-2.779	4.188	8.76%

Table S4. Si-C bond lengths and Merz-Kollmann (MK) charges on the Si-atom and adjacent carbons.

XCF/basis set	Bond length (Å)			MK charges (e)			
	C1-Si	C2-Si	C3-Si	C1	C2	C3	Si
ωB97XD/6-31G(d)	1.98894	1.98900	1.98895	0.116	0.110	0.081	-0.222
ωB97XD/6-311+G(d,p)	1.98812	1.98799	1.98801	0.069	0.093	0.064	-0.162
ωB97XD/DF2TZVP	1.97993	1.97987	1.97976	0.053	0.108	0.107	-0.191
M062X/6-31G(d)	2.00155	2.00156	2.00131	0.047	0.047	0.061	-0.142
M062X/6-311+G(d,p)	2.00052	2.00063	2.00038	0.031	0.060	0.035	-0.092
M062X/6-311++G(d,p)	2.00052	2.00063	2.00038	0.031	0.060	0.035	-0.092
M062X/DF2TZVP	1.99038	1.99058	1.99057	0.085	0.090	0.028	-0.143
M06L/6-31G(d)	1.99147	1.99131	1.9914	0.032	0.082	0.075	-0.174
M06L/6-311+G(d,p)	1.99006	1.99000	1.98985	0.075	0.023	0.070	-0.142
M06L/DF2TZVP	1.98098	1.98094	1.98089	0.048	0.075	0.047	-0.159
B3LYP/6-31G(d)	2.00814	2.00839	2.00871	0.106	0.105	0.022	-0.222
B3LYP/6-311+G(d,p)	2.00745	2.00737	2.00734	0.054	0.044	0.031	-0.112
B3LYP/DF2TZVP	1.99733	1.9974	1.99736	0.072	0.048	0.051	-0.131

Table S5. Adsorption energies (in eV), E_{ads}, and adsorption energy with and without BSSE corrections, $E_{ads}\,(BSSE)$, for the dimers formed between C_{20} fullerenes with CO, CO_2 and N_2 molecules calculated with the theoretical levels ωB97XD /6-31G(d) and ωB97XD/6-311+G(d,p).

Molecules	ωB97XD/6-31G(d)			ωB97XD/6-311+G(d.p)		
	BSSE	E_{ads}	$E_{ads}(BSSE)$	BSSE	E_{ads}	$E_{ads}(BSSE)$
C_{20}-CO	0.040	-0.080	-0.040	0.023	-0.065	-0.042
C_{20}-CO_2	0.045	-0.110	-0.066	0.029	-0.104	-0.075
C_{20}-N_2	0.034	-0.076	-0.042	0.024	-0.066	-0.042

Table S6. Externally oriented electric field effect on the BSSE correction, adsorption energies, E_{ads}, and BSSE corrected, $E_{ads}(BSSE)$, for the interaction of C_{20} and $C_{19}Si$ fullerenes with the CO, CO_2 and N_2 molecules as obtained at the ωB97XD/6-31G(d) and ωB97XD/6-311+G(d,p) levels of theory. Adjusted adsorption energy, $E_{ads}(fit)$ and the R-squared coefficient of determination, R^2, are also presented. All energies in eV.

ωB97XD/6-31G(d)					ωB97XD/6-311+G(d.p)				
$C_{19}Si$-CO									
F	BSSE	E_{ads}	$E_{ads}(BSSE)$	$E_{ads}(fit)$	F	BSSE	E_{ads}	$E_{ads}(BSSE)$	$E_{ads}(fit)$
0.000	0.134	-0.837	-0.703	-0.650	0.000	0.049	-0.787	-0.738	-0.677
0.001	0.134	-0.884	-0.749	-0.717	0.001	0.049	-0.838	-0.788	-0.749
0.005	0.140	-1.097	-0.957	-0.987	0.005	0.050	-1.060	-1.009	-1.039
0.010	0.148	-1.409	-1.261	-1.323	0.010	0.052	-1.380	-1.328	-1.401
0.015	0.157	-1.766	-1.609	-1.660	0.015	0.052	-1.746	-1.694	-1.763
0.020	0.168	-2.157	-1.989	-1.997	0.020	0.054	-2.159	-2.105	-2.125
0.025	0.180	-2.581	-2.400	-2.334	0.025	0.053	-2.631	-2.579	-2.488
R^2	0.994				R^2	0.991			
$C_{19}Si$-CO_2									
F	BSSE	E_{ads}	$E_{ads}(BSSE)$	$E_{ads}(fit)$	F	BSSE	E_{ads}	$E_{ads}(BSSE)$	$E_{ads}(fit)$
0.000	0.027	-0.095	-0.068	-0.076	0.000	0.015	-0.092	-0.077	-0.083
0.001	0.040	-0.121	-0.081	-0.075	0.001	0.023	-0.109	-0.087	-0.082
0.005	0.045	-0.130	-0.085	-0.081	0.005	0.026	-0.118	-0.091	-0.09
0.010	0.052	-0.162	-0.110	-0.110	0.010	0.029	-0.153	-0.124	-0.125
0.015	0.062	-0.223	-0.161	-0.162	0.015	0.032	-0.225	-0.194	-0.189
0.020	0.076	-0.310	-0.234	-0.238	0.020	0.034	-0.307	-0.272	-0.280
0.025	0.093	-0.432	-0.339	-0.337	0.025	0.036	-0.438	-0.402	-0.399
R^2	0.998				R^2	0.998			
$C_{19}Si$-N_2									
F	BSSE	E_{ads}	$E_{ads}(BSSE)$	$E_{ads}(fit)$	F	BSSE	E_{ads}	$E_{ads}(BSSE)$	$E_{ads}(fit)$
0.000	0.134	-0.837	-0.017	-0.031	0.000	0.024	-0.069	-0.045	-0.044
0.001	0.134	-0.884	-0.045	-0.034	0.001	0.026	-0.072	-0.046	-0.046
0.005	0.140	-1.097	-0.054	-0.048	0.005	0.026	-0.082	-0.056	-0.057
0.010	0.148	-1.409	-0.072	-0.072	0.010	0.027	-0.104	-0.077	-0.078
0.015	0.157	-1.766	-0.097	-0.101	0.015	0.029	-0.143	-0.114	-0.109
0.020	0.168	-2.157	-0.132	-0.135	0.020	0.030	-0.177	-0.146	-0.150
0.025	0.180	-2.581	-0.177	-0.174	0.025	0.032	-0.233	-0.201	-0.200

R^2	0.980	R^2	0.998

Table S7. Energies (in eV) of the HOMO, E_H, LUMO, E_L and the gap between them, E_{HL}, calculated with the theoretical levels ωB97XD/6-31G(d) and ωB97XD/6-311+G(d,p), for the dimers.. The percentual change in the fullerene energy gap after adsorbing the gas, in relation to the fullerene energy gap before adsorption, is represented by ΔE_{HL} (in %). Positive values of ΔE_{HL} indicate an increase in the gap, negative values indicate a reduction.

Dimers	ωB97XD /6-31G(d)				ωB97XD /6-311+G(d,p)			
	$F = 0.000\ a.u.$							
	E_H	E_L	E_{HL}	ΔE_{HL}	E_H	E_L	E_{HL}	ΔE_{HL}
C_{20}-CO	-7.045	-1.580	5.465	0.130	-7.355	-1.957	5.397	0.050
C_{20}-CO_2	-7.072	-1.607	5.465	0.145	-7.405	-2.011	5.394	-0.020
C_{20}-N_2	-7.039	-1.579	5.461	0.060	-7.356	-1.958	5.397	0.050
Dimers	$F = 0.000\ a.u.$							
	E_H	E_L	E_{HL}	ΔE_{HL}	E_H	E_L	E_{HL}	ΔE_{HL}
C_{19}Si-CO	-6.888	-2.256	4.632	-20.212	-7.188	-2.609	4.579	-19.966
C_{19}Si-CO_2	-7.516	-1.739	5.776	-0.497	-7.740	-2.055	5.684	-0.652
C_{19}Si-N_2	-7.534	-1.733	5.801	-0.080	-7.760	-2.048	5.712	-0.171
Dimers	$F = 0.001\ a.u.$							
	E_H	E_L	E_{HL}	ΔE_{HL}	E_H	E_L	E_{HL}	ΔE_{HL}
C_{19}Si-CO	-7.257	-2.637	4.620	-20.418	-7.563	-2.981	4.581	-19.928
C_{19}Si-CO_2	-7.791	-2.031	5.760	-0.778	-7.759	-2.093	5.666	-0.975
C_{19}Si-N_2	-7.734	-1.949	5.785	-0.351	-7.766	-2.072	5.694	-0.485
Dimers	$F = 0.005\ a.u.$							
	E_H	E_L	E_{HL}	ΔE_{HL}	E_H	E_L	E_{HL}	ΔE_{HL}
C_{19}Si-CO	-8.741	-4.125	4.616	-20.488	-9.067	-4.491	4.576	-20.023
C_{19}Si-CO_2	-8.889	-3.201	5.688	-2.030	-9.131	-3.543	5.588	-2.340
C_{19}Si-N_2	-8.522	-2.814	5.708	-1.683	-8.767	-3.154	5.613	-1.893
Dimers	$F = 0.010\ a.u.$							
	E_H	E_L	E_{HL}	ΔE_{HL}	E_H	E_L	E_{HL}	ΔE_{HL}
C_{19}Si-CO	-10.589	-6.014	4.575	-21.192	-10.959	-6.416	4.543	-20.594
C_{19}Si-CO_2	-10.279	-4.673	5.605	-3.446	-10.556	-5.051	5.504	-3.794
C_{19}Si-N_2	-9.504	-3.896	5.607	-3.412	-9.783	-4.275	5.507	-3.748
Dimers	$F = 0.015\ a.u.$							
	E_H	E_L	E_{HL}	ΔE_{HL}	E_H	E_L	E_{HL}	ΔE_{HL}
C_{19}Si-CO	-12.431	-7.920	4.511	-22,297	-12,869	-8,377	4,492	-21,498
C_{19}Si-CO_2	-11.657	-6.143	5.514	-5,016	-8,153	-2,720	5,433	-5,046
C_{19}Si-N_2	-10.485	-4.980	5.505	-5,166	-7,904	-2,479	5,425	-5,175
Dimers	$F = 0.020\ a.u.$							
	E_H	E_L	E_{HL}	ΔE_{HL}	E_H	E_L	E_{HL}	ΔE_{HL}
C_{19}Si-CO	-14.298	-9.830	4.468	-23.033	-14.808	-10.373	4.435	-22.492
C_{19}Si-CO_2	-13.036	-7.607	5.429	-6.477	-13.433	-8.136	5.297	-7.414
C_{19}Si-N_2	-11.475	-6.065	5.410	-6.807	-11.869	-6.595	5.274	-7.815

Dimers	$F = 0.025\ a.u.$							
	E_H	E_L	E_{HL}	ΔE_{HL}	E_H	E_L	E_{HL}	ΔE_{HL}
$C_{19}Si$-CO	-16.191	-11.740	4.451	-23.326	-16.768	-12.492	4.276	-25.264
$C_{19}Si$-CO_2	-14.439	-9.066	5.373	-7.439	-14.956	-10.362	4.594	-19.703
$C_{19}Si$-N_2	-12.484	-7.155	5.330	-8.194	-12.996	-8.488	4.508	-21.212

Table S8. Topological parameters calculated using the Quantum Theory of Atoms in Molecules (QTAIM) with the theoretical level ωB97XD /6-31G(d). Results are presented for all dimers formed by C_{20} and $C_{19}Si$ fullerenes with adsorbed gas molecules. The results were obtained for all electric field values and are all presented in atomic units.

Dimers	**ωB97XD/6-31G(d)**							
	$F = 0.000\ a.u.$							
	BCP	Bond	$\rho_{BCP} \times 10^{-3}$	$\nabla^2\rho_{BCP} \times 10^{-2}$	$H_{BCP} \times 10^{-4}$	$G_{BCP} \times 10^{-3}$	$V_{BCP} \times 10^{-3}$	$\lvert G_{BCP}/V_{BCP}\rvert$
C_{20}-CO	1	C-C	3.704	1.239	6.475	2.451	-1.804	1.359
	2	C-O	2.760	1.132	5.118	2.317	-1.806	1.283
C_{20}-CO_2	1	C-O	2.926	1.225	4.988	2.563	-2.064	1.242
	2	C-O	2.458	1.122	4.738	2.331	-1.857	1.255
	3	C-O	2.441	1.094	4.170	2.317	-1.900	1.220
C_{20}-N_2	1	C-N	3.564	1.146	4.610	2.404	-1.943	1.237
	2	C-N	3.677	1.184	4.532	2.507	-2.054	1.221
$C_{19}Si$-CO	1	Si-C	18.256	13.592	-14.977	35.478	-36.976	0.959
	2	Si-C	16.693	13.265	-9.297	35.533	-37.084	0.958
	3	Si-C	18.351	13.593	-15.515	34.092	-35.022	0.973
	4	Si-C	16.969	35.720	137.366	75.564	-61.827	1.222
	5		16.968	35.240	133.473	74.753	-61.406	1.2174
	6		16.950	33.938	123.195	72.525	-60.205	1.2046
	7		15.970	27.519	79.730	60.825	-52.852	1.1509
$C_{19}Si$-CO_2	1	Si-O	3.623	1.257	4.928	2.649	-2.156	1.2285
	2	Si-O	3.906	1.301	8.257	2.428	-1.602	1.5155
$C_{19}Si$-N_2	1	Si-N	1.955	0.557	3.054	1.087	-0.782	1.391
**Dimers**	$F = 0.001\ a.u.$							
	BCP	Bond	$\rho_{BCP} \times 10^{-3}$	$\nabla^2\rho_{BCP} \times 10^{-2}$	$H_{BCP} \times 10^{-4}$	$G_{BCP} \times 10^{-3}$	$V_{BCP} \times 10^{-3}$	$\lvert G_{BCP}/V_{BCP}\rvert$
$C_{19}Si$-CO	1	Si-C	17.948	13.307	-14.384	34.705	-36.144	0.960
	2	Si-C	17.850	13.302	-13.867	34.641	-36.027	0.961
	3	Si-C	16.473	12.987	-9.191	33.387	-34.306	0.973
	4		16.504	33.454	122.812	71.355	-59.073	1.2079
	5		15.669	27.605	82.555	60.757	-52.501	1.1572
$C_{19}Si$-CO2	1	Si-O	5.278	1.743	2.901	4.068	-3.778	1.0768
$C_{19}Si$-N2	1	C-N	3.342	1.124	4.152	2.394	-1.979	1.210
	2	Si-N	3.464	1.027	3.293	2.239	-1.9010	1.172
**Dimers**	$F = 0.005\ a.u.$							
	BCP	Bond	$\rho_{BCP} \times 10^{-3}$	$\nabla^2\rho_{BCP} \times 10^{-2}$	$H_{BCP} \times 10^{-4}$	$G_{BCP} \times 10^{-3}$	$V_{BCP} \times 10^{-3}$	$\lvert G_{BCP}/V_{BCP}\rvert$
$C_{19}Si$-CO	1	Si-C	17.254	13.077	-11.884	33.881	-35.070	0.966
	2	Si-C	17.202	13.079	-11.575	33.856	-35.013	0.967

	3	Si-C	16.017	12.768	-7.9833	32.719	-33.517	0.976
	4	Si-C	16.011	34.688	136.235	73.099	-59.476	1.229
C_{19}Si-CO2	1	C-O	3.390	1.521	6.239	3.178	-0.624	5.093
	2	Si-O	5.740	1.932	2.848	4.545	-4.260	1.067
C_{19}Si-N2	1	C-N	3.624	1.192	4.301	2.551	-2.121	1.203
	2	Si-N	3.622	1.113	3.541	2.427	-2.072	1.171
**Dimers**	$F = 0.010\ a.u.$							
	BCP	Bond	$\rho_{BCP} \times 10^{-3}$	$\nabla^2\rho_{BCP} \times 10^{-2}$	$H_{BCP} \times 10^{-4}$	$G_{BCP} \times 10^{-3}$	$V_{BCP} \times 10^{-3}$	$\lvert G_{BCP}/V_{BCP} \rvert$
	1	Si-C	16.202	12.805	-7.832	32.795	-33.578	0.977
	2	Si-C	16.726	12.995	-9.367	33.423	-34.360	0.973
C_{19}Si-CO	3	Si-C	15.465	12.554	-5.991	31.985	-32.584	9.816
	4		15.313	28.936	96.422	62.697	-53.054	1.182
	5		14.762	26.454	80.775	58.058	-49.981	1.162
C_{19}Si-CO2	1		3.925	1.760	7.226	3.677	-2.955	1.245
	2		6.528	2.172	1.915	5.210	-5.048	1.038
C_{19}Si-N2	1	C-N	3.950	1.294	4.499	2.786	-2.336	1.193
	2	Si-N	4.064	1.292	3.853	2.845	-2.460	1.157
**Dimers**	$F = 0.015\ a.u.$							
	BCP	Bond	$\rho_{BCP} \times 10^{-3}$	$\nabla^2\rho_{BCP} \times 10^{-2}$	$H_{BCP} \times 10^{-4}$	$G_{BCP} \times 10^{-3}$	$V_{BCP} \times 10^{-3}$	$\lvert G_{BCP}/V_{BCP} \rvert$
C_{19}Si-CO	1	Si-C	66.488	24.612	-246.685	86.198	-110.866	0.778
C_{19}Si-CO_2	1	C-O	4.174	1.920	7.843	4.017	-7.843	5.121
	2	Si-O	8.587	2.718	-1.205	6.917	-7.037	0.983
C_{19}Si-N2	1	C-N	2.982	1.195	5.559	2.431	-1.876	1.2964
	2	Si-N	5.814	1.913	4.681	4.315	-3.846	1.1217
**Dimers**	$F = 0.020\ a.u.$							
	BCP	Bond	$\rho_{BCP} \times 10^{-3}$	$\nabla^2\rho_{BCP} \times 10^{-2}$	$H_{BCP} \times 10^{-4}$	$G_{BCP} \times 10^{-3}$	$V_{BCP} \times 10^{-3}$	$\lvert G_{BCP}/V_{BCP} \rvert$
C_{19}Si-CO	1	Si-C	75.218	18.519	-317.233	78.021	-109.74	0.711
C_{19}Si-CO2	1	Si-O	12.480	2.993	-2.139	7.696	-7.910	0.973
C_{19}Si-N2	1	Si-N	8.297	2.100	4.859	4.764	-4.278	1.114
**Dimers**	$F = 0.025\ a.u.$							
	BCP	Bond	$\rho_{BCP} \times 10^{-3}$	$\nabla^2\rho_{BCP} \times 10^{-2}$	$H_{BCP} \times 10^{-4}$	$G_{BCP} \times 10^{-3}$	$V_{BCP} \times 10^{-3}$	$\lvert G_{BCP}/V_{BCP} \rvert$
C_{19}Si-CO	1	Si-C	77.475	17.822	-340.719	78.628	-112.699	0.698
C_{19}Si-CO2	1	Si-O	18.086	3.699	-10.581	10.306	-11.364	0.907
C_{19}Si-N2	1	Si-N	10.488	2.481	4.123	5.710	-53.776	1.0767

Table S9. Topological parameters calculated using the Quantum Theory of Atoms in Molecules (QTAIM) with the theoretical level ωB97XD /6-311+G(d,p). Results are presented for all dimers formed by C_{20} and $C_{19}Si$ fullerenes with adsorbed gas molecules. The results were obtained for all electric field values and are all presented in atomic units.

Dimers	ωB97XD/6-311+G(d.p)							
	$F = 0.000\ a.u.$							
	BCP	Bond	$\rho_{BCP} \times 10^{-3}$	$\nabla^2\rho_{BCP} \times 10^{-2}$	$H_{BCP} \times 10^{-4}$	$G_{BCP} \times 10^{-3}$	$V_{BCP} \times 10^{-3}$	$\lvert G_{BCP}/V_{BCP}$
C_{20}-CO	1	C-C	3.686	1.054	6.371	1.998	-1.361	1.468
	2	C-O	2.736	0.978	5.130	1.932	-1.419	1.361
C_{20}-CO_2	1	C-C	4.766	1.884	11.374	3.572	-2.434	1.467
C_{20}-N_2	1	C-N	2.866	0.962	5.162	1.888	-1.372	1.376
	2	C-N	2.635	0.872	4.617	1.718	-1.256	1.367
$C_{19}Si$-CO	1	Si-C	70.630	20.211	-276.945	78.223	-105.91	0.738
$C_{19}Si$-CO_2	1	Si-O	5.744	1.625	7.969	3.266	-2.470	1.323
$C_{19}Si$-N_2	1	C-N	3.078	1.026	5.654	2.000	-1.435	1.394
	2	Si-N	4.126	1.135	4.793	2.360	-1.880	1.255
Dimers	$F = 0.001\ a.u.$							
	BCP	Bond	$\rho_{BCP} \times 10^{-3}$	$\nabla^2\rho_{BCP} \times 10^{-2}$	$H_{BCP} \times 10^{-4}$	$G_{BCP} \times 10^{-3}$	$V_{BCP} \times 10^{-3}$	$\lvert G_{BCP}/V_{BCP}$
$C_{19}Si$-CO	1	Si-C	70.630	20.211	-276.945	78.223	-105.917	0.738
$C_{19}Si$-CO_2	1	Si-O	6.177	1.633	4.400	3.643	-3.203	1.137
$C_{19}Si$-N_2	1	C-N	3.665	1.215	6.606	2.376	-1.716	1.385
	2	Si-N	4.514	1.253	5.124	2.620	-2.107	1.243
Dimers	$F = 0.005\ a.u.$							
	BCP	Bond	$\rho_{BCP} \times 10^{-3}$	$\nabla^2\rho_{BCP} \times 10^{-2}$	$H_{BCP} \times 10^{-4}$	$G_{BCP} \times 10^{-3}$	$V_{BCP} \times 10^{-3}$	$\lvert G_{BCP}/V_{BCP}$
$C_{19}Si$-CO	1	Si-C	71.154	19.405	-285.140	77.027	-105.541	0.730
$C_{19}Si$-CO_2	1	C-O	3.922	1.523	8.174	2.990	-2.173	1.376
	2	Si-O	6.170	1.665	3.751	3.787	-3.412	1.110
$C_{19}Si$-N_2	1	C-N	3.408	1.135	6.285	2.208	-1.579	1.398
	2	Si-N	4.519	1.254	5.144	2.622	-2.107	1.244
Dimers	$F = 0.010\ a.u.$							
	BCP	Bond	$\rho_{BCP} \times 10^{-3}$	$\nabla^2\rho_{BCP} \times 10^{-2}$	$H_{BCP} \times 10^{-4}$	$G_{BCP} \times 10^{-3}$	$V_{BCP} \times 10^{-3}$	$\lvert G_{BCP}/V_{BCP}$
$C_{19}Si$-CO	1	Si-C	73.074	18.976	-303.144	77.755	-108.070	0.719
$C_{19}Si$-CO_2	1	C-O	4.963	1.969	10.203	3.902	-2.881	1.354
	2	Si-O	6.909	1.945	4.489	4.414	-3.965	1.113
$C_{19}Si$-N_2	1	C-N	3.812	1.279	7.106	2.487	-1.776	1.400
	2	Si-N	5.310	1.459	5.588	3.088	-2.529	1.221
Dimers	$F = 0.015\ a.u.$							
	BCP	Bond	$\rho_{BCP} \times 10^{-3}$	$\nabla^2\rho_{BCP} \times 10^{-2}$	$H_{BCP} \times 10^{-4}$	$G_{BCP} \times 10^{-3}$	$V_{BCP} \times 10^{-3}$	$\lvert G_{BCP}/V_{BCP}$
$C_{19}Si$-CO	1	Si-C	75.200	18.480	-323.587	78.559	-110.918	0.708
$C_{19}Si$-CO_2	1	C-O	5.041	2.053	11.121	4.020	-2.908	1.382

Dimers	BCP	Bond	$\rho_{BCP} \times 10^{-3}$	$\nabla^2\rho_{BCP} \times 10^{-2}$	$H_{BCP} \times 10^{-4}$	$G_{BCP} \times 10^{-3}$	$V_{BCP} \times 10^{-3}$	$\|G_{BCP}/V_{BCP}$
	2	Si-O	8.960	2.422	4.184	5.638	-5.219	1.080
$C_{19}Si$-N_2	1	C-N	4.061	1.393	7.806	2.703	-1.922	1.406
	2	Si-N	6.513	1.741	6.012	3.752	-3.150	1.191
			$F = 0.020\ a.u.$					
**Dimers**	BCP	Bond	$\rho_{BCP} \times 10^{-3}$	$\nabla^2\rho_{BCP} \times 10^{-2}$	$H_{BCP} \times 10^{-4}$	$G_{BCP} \times 10^{-3}$	$V_{BCP} \times 10^{-3}$	$\|G_{BCP}/V_{BCP}$
$C_{19}Si$-CO	1	Si-C	78.138	18.319	-349.540	80.751	-115.705	0.698
$C_{19}Si$-CO_2	1	Si-O	12.880	3.2435	2.4203	7.867	-7.625	1.032
$C_{19}Si$-N_2	1	C-N	4.209	1.522	8.479	2.957	-2.109	1.402
	2	Si-N	8.899	2.281	6.614	5.040	-4.379	1.151
			$F = 0.025\ a.u.$					
**Dimers**	BCP	Bond	$\rho_{BCP} \times 10^{-3}$	$\nabla^2\rho_{BCP} \times 10^{-2}$	$H_{BCP} \times 10^{-4}$	$G_{BCP} \times 10^{-3}$	$V_{BCP} \times 10^{-3}$	$\|G_{BCP}/V_{BCP}$
$C_{19}Si$-CO	1	Si-C	80.146	17.195	-373.751	80.364	-1.17.739	0.682
$C_{19}Si$-CO_2	1	Si-O	19.799	4.395	-4.569	11.445	-11.902	0.962
$C_{19}Si$-N_2	1	Si-N	11.871	28.437	6.329	6.476	-5.843	1.108

Table S10. Critical bond points, BCP, isosurfaces and scatter plots for the dimers formed by C_{20} and $C_{19}Si$ fullerenes with the adsorbed gas molecules. The results were obtained for all electric field values using both the theoretical level ωB97XD/6-31G(d) and ωB97XD/6-311+G(d,p).

**ωB97XD/6-31G(d)**	**ωB97XD/6-311+G(d,p)**
**C_{20}-CO**<br>$F = 0.000\ a.u.$<br>$E_{ads}(BSSE) = -0.04027087\ eV$	**C_{20}-CO**<br>$F = 0.000\ a.u.$<br>$E_{ads}(BSSE) = -0.04215341\ eV$
QTAIM	
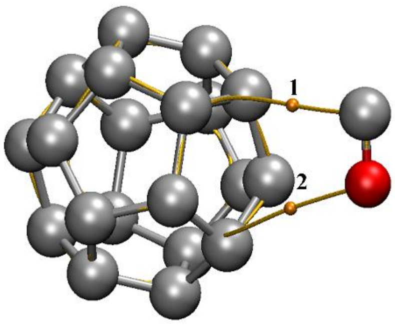	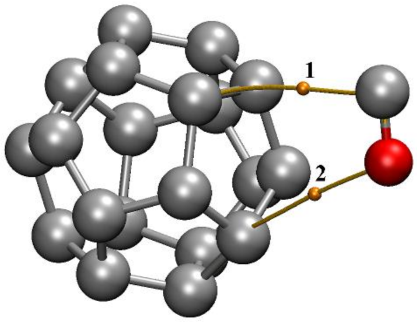
RDG	

<table>
<tr><td>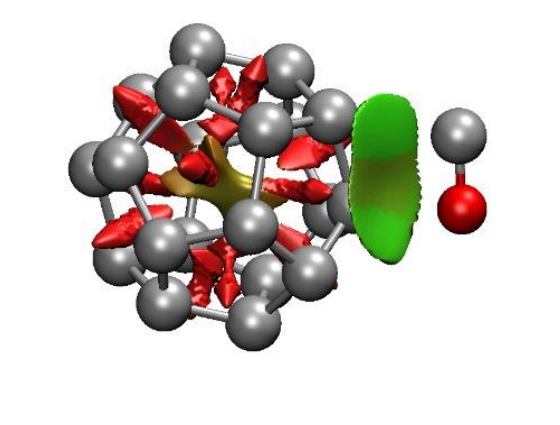</td><td>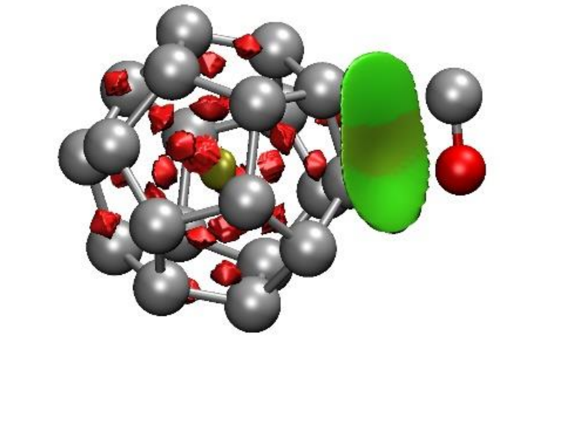</td></tr>
<tr><td colspan="2">Scatter graph</td></tr>
<tr><td>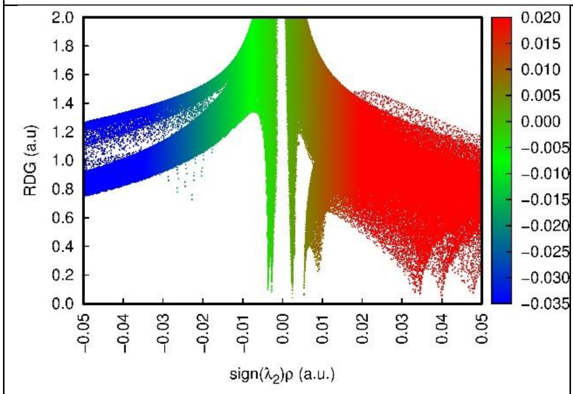
</td><td>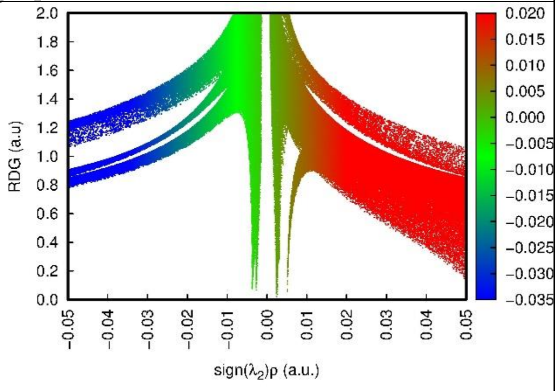
</td></tr>
<tr><td>C20-CO2
$F = 0.000\ a.u.$
$E_{ads}(BSSE) = -0.06556463\ eV$</td><td>C20-CO2
$F = 0.000\ a.u.$
$E_{ads}(BSSE) = -0.07502922\ eV$</td></tr>
<tr><td colspan="2">QTAIM</td></tr>
<tr><td>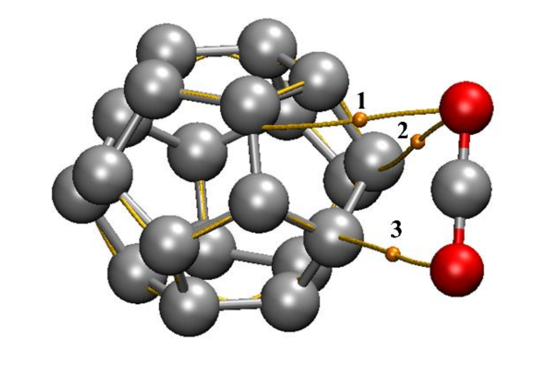
</td><td>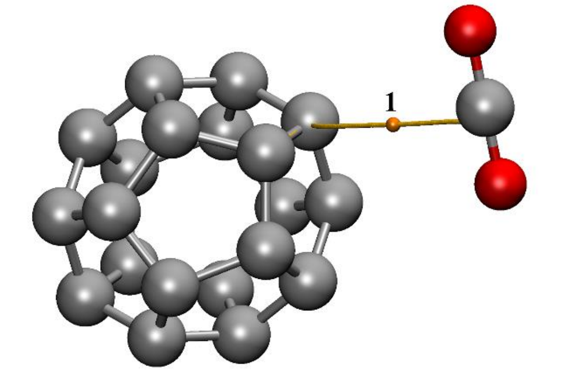
</td></tr>
<tr><td colspan="2">RDG</td></tr>
<tr><td>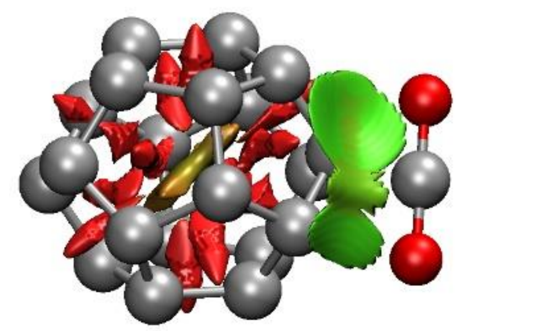</td><td>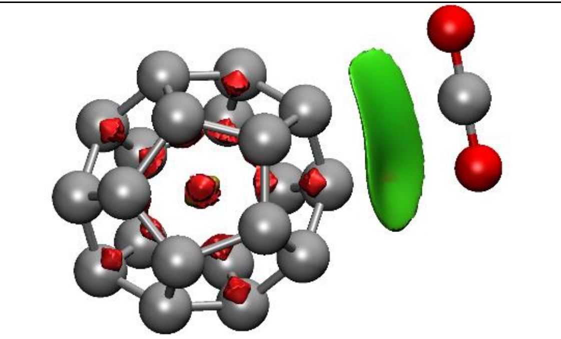</td></tr>
<tr><td colspan="2">Scatter graph</td></tr>
</table>

<table>
<tr><td>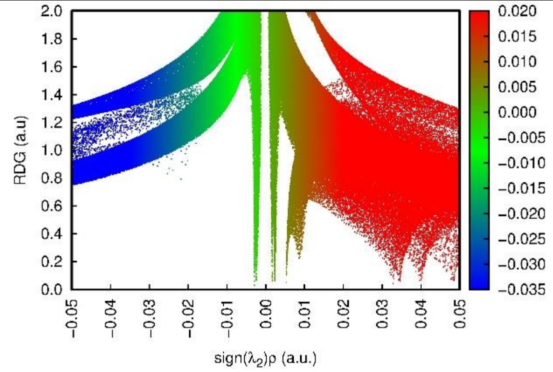
</td><td>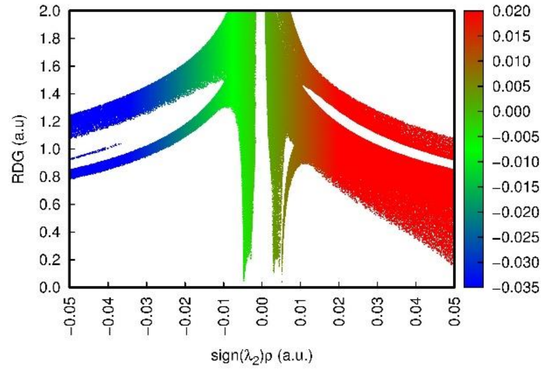
</td></tr>
<tr><td>C_{20}-N_2
$F = 0.000\ a.u.$
$E_{ads}(BSSE) = \ -0.04191301 eV$</td><td>$C_{20}$-$N_2$
$F = 0.000\ a.u.$
$E_{ads}(BSSE) = -0.04214772\ eV$</td></tr>
<tr><td colspan="2">QTAIM</td></tr>
<tr><td>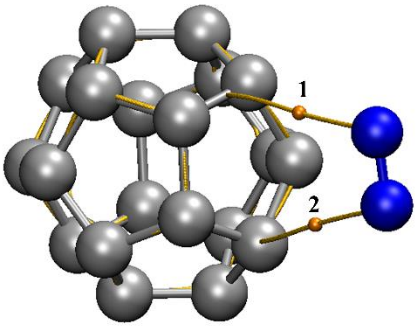
</td><td>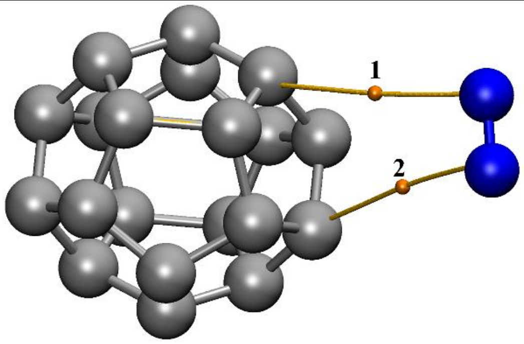
</td></tr>
<tr><td colspan="2">RDG</td></tr>
<tr><td>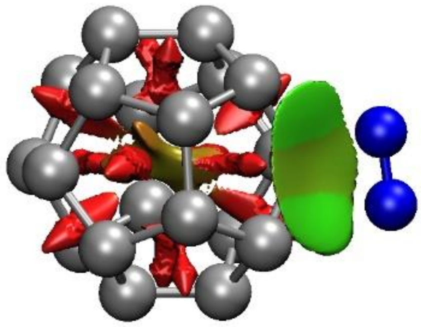</td><td>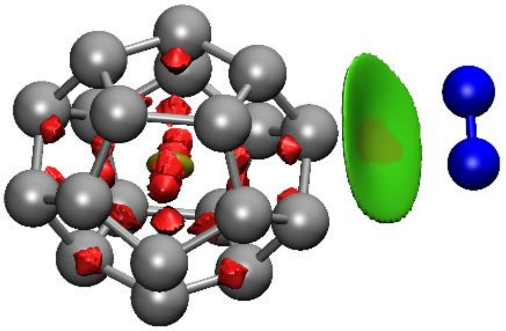</td></tr>
<tr><td colspan="2">Scatter graph</td></tr>
<tr><td>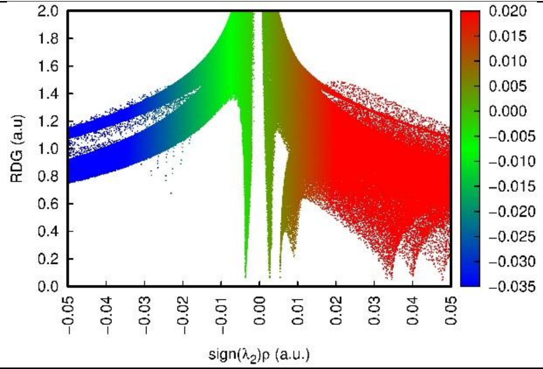
</td><td>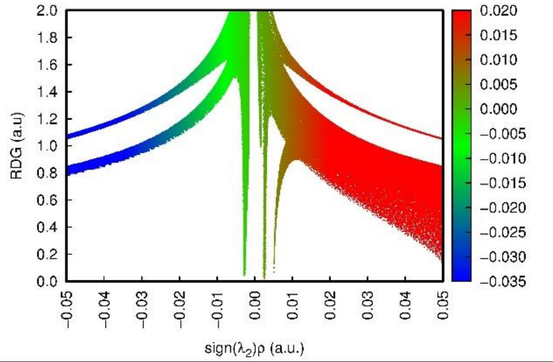
</td></tr>
<tr><td>C_{19}Si-CO
$F = 0.000\ a.u.$
$E_{ads}(BSSE) = \ -0.70264080\ eV$</td><td>$C_{19}$Si-CO
$F = 0.000\ a.u.$
$E_{ads}(BSSE) = \ -0.73799673\ eV$</td></tr>
<tr><td colspan="2">QTAIM</td></tr>
</table>

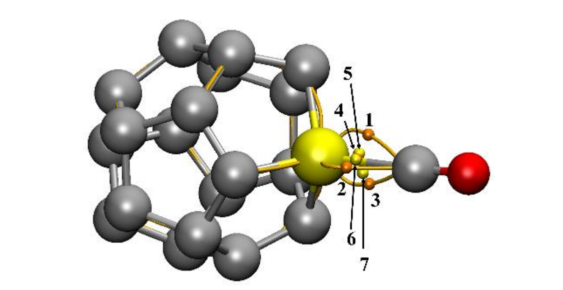

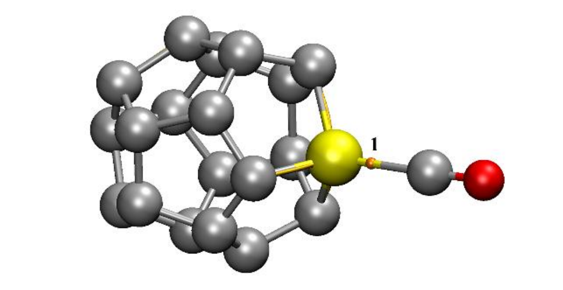

RDG

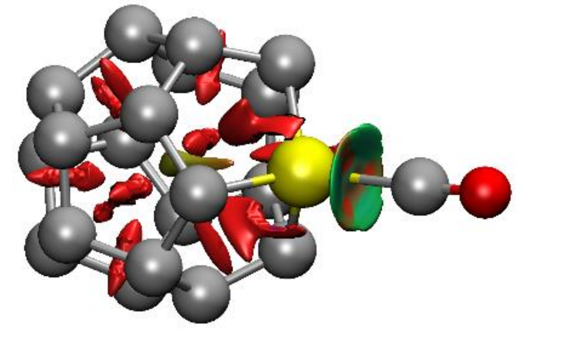

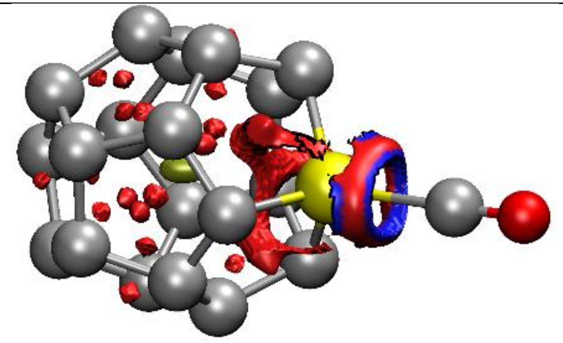

Scatter graph

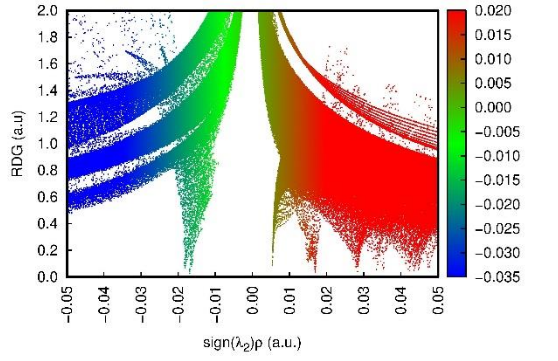

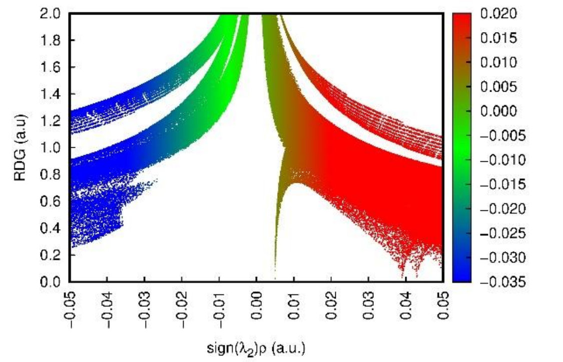

C_{19}Si-CO_2
$F = 0.000\ a.u.$
$E_{ads}(BSSE) = -0.06827564\ eV$

C_{19}Si-CO_2
$F = 0.000\ a.u.$
$E_{ads}(BSSE) = -0.07742641\ eV$

QTAIM

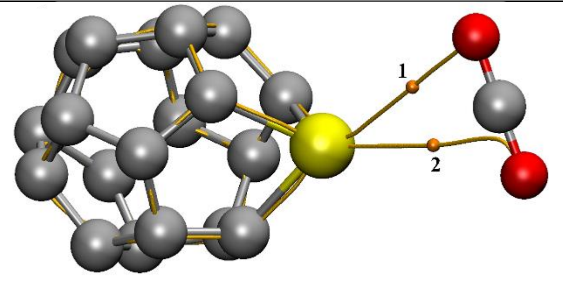

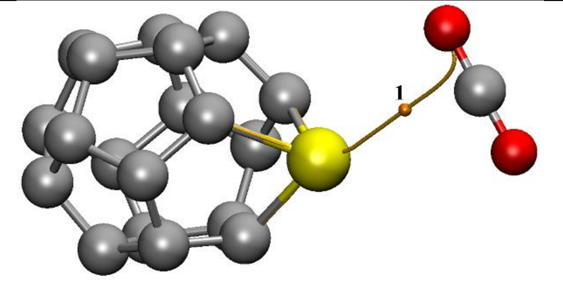

RDG

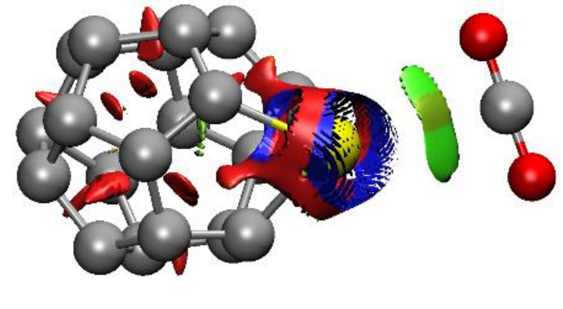

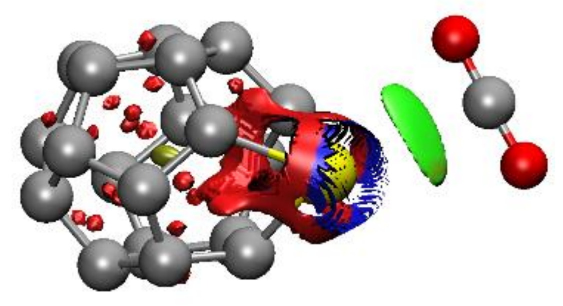

<table>
<tr><td colspan="2">Scatter graph</td></tr>
<tr><td>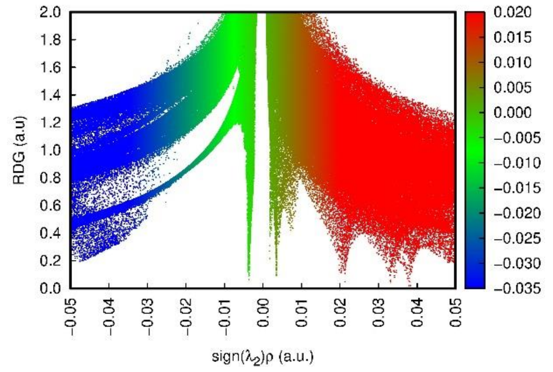
</td><td>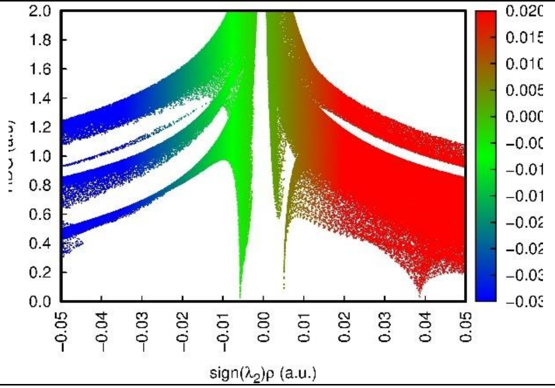
</td></tr>
<tr><td>C_{19}Si-N_2
$F = 0.000\ a.u.$
$E_{ads}(BSSE) = -0.01722895\ eV$</td><td>$C_{19}$Si-$N_2$
$F = 0.000\ a.u.$
$E_{ads}(BSSE) = -0.04477245\ eV$</td></tr>
<tr><td colspan="2">QTAIM</td></tr>
<tr><td>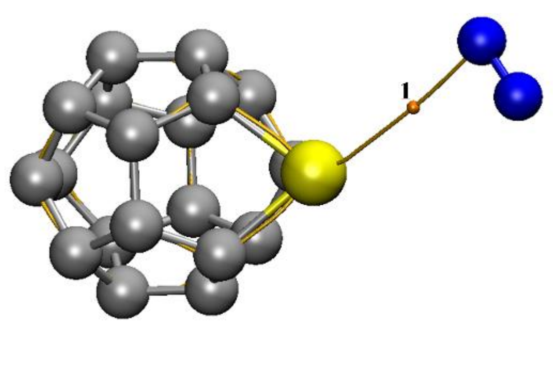
</td><td>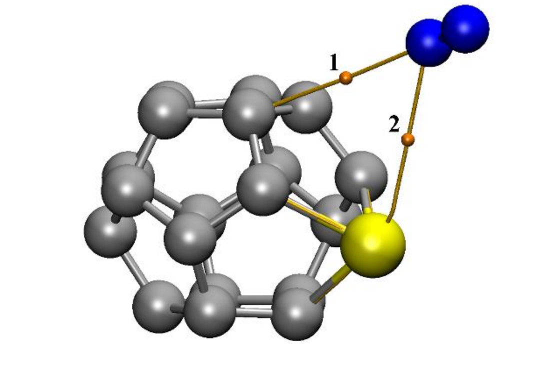
</td></tr>
<tr><td colspan="2">RDG</td></tr>
<tr><td>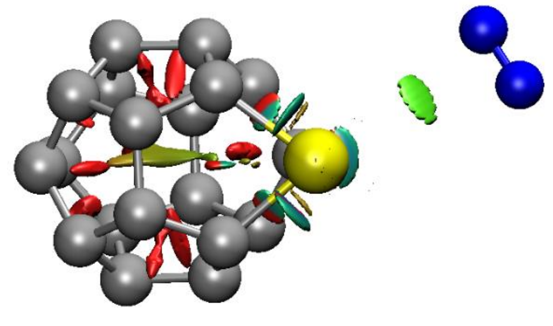</td><td>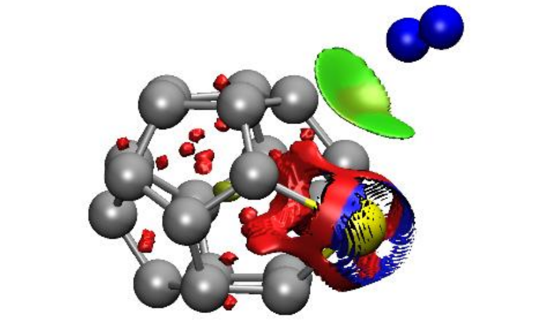</td></tr>
<tr><td colspan="2">Scatter graph</td></tr>
<tr><td>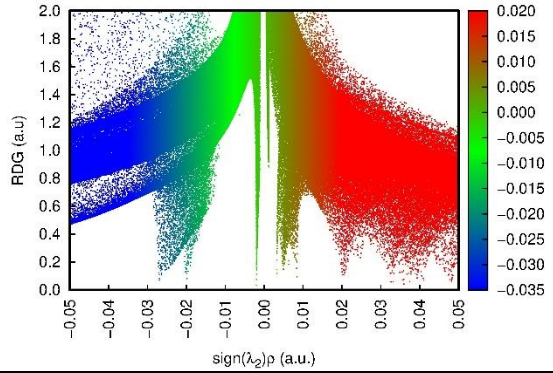
</td><td>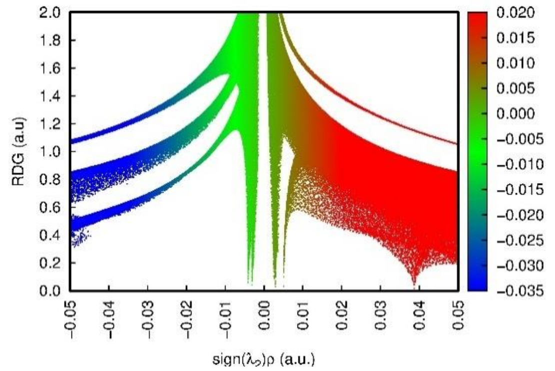
</td></tr>
<tr><td>C_{19}Si-CO
$F = 0.001\ a.u.$
$E_{ads}(BSSE) = -0.74916933\ eV$</td><td>$C_{19}$Si-CO
$F = 0.001\ a.u.$
$E_{ads}(BSSE) = -0.78821735\ eV$</td></tr>
</table>

<table>
<tr><td colspan="2">QTAIM</td></tr>
<tr><td>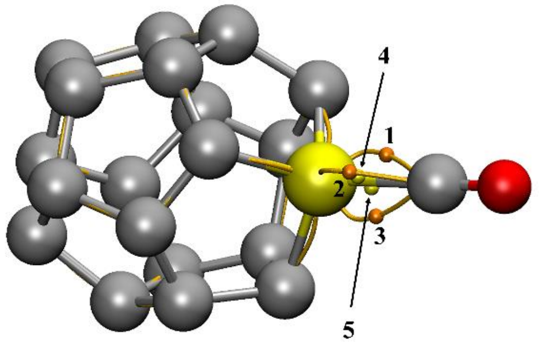
</td><td>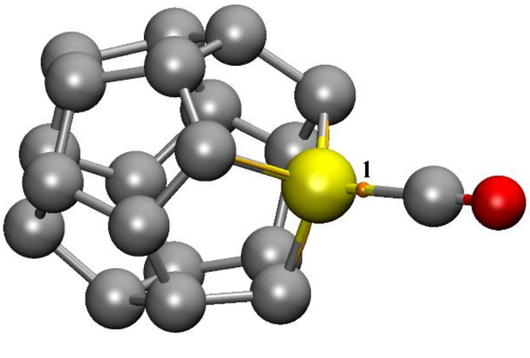
</td></tr>
<tr><td colspan="2">RDG</td></tr>
<tr><td>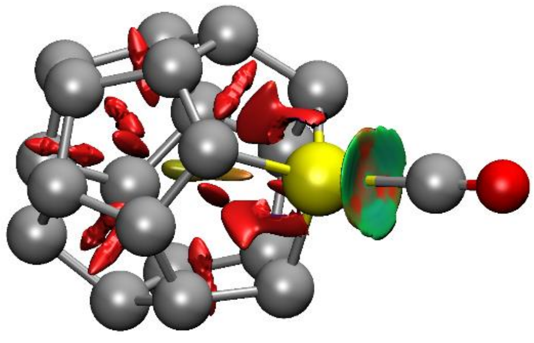</td><td>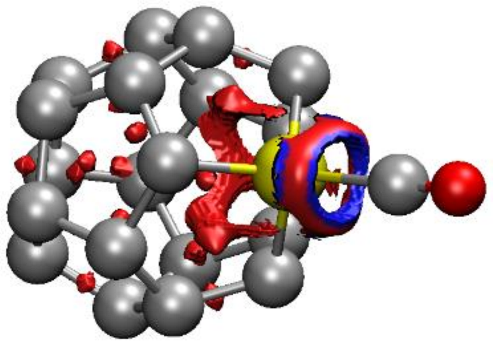</td></tr>
<tr><td colspan="2">Scatter graph</td></tr>
<tr><td>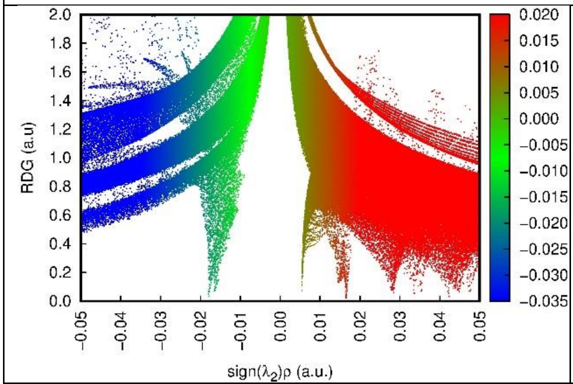
</td><td>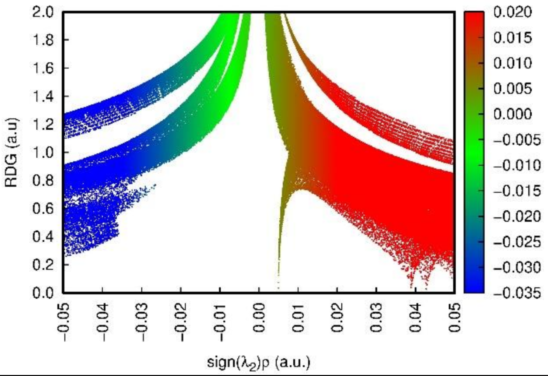
</td></tr>
<tr><td>$C_{19}Si$-CO_2
$F = 0.001\ a.u.$
$E_{ads}(BSSE) = -0.08110533\ eV$</td><td>$C_{19}Si$-$CO_2$
$F = 0.001\ a.u.$
$E_{ads}(BSSE) = -0.08655367\ eV$</td></tr>
<tr><td colspan="2">QTAIM</td></tr>
<tr><td>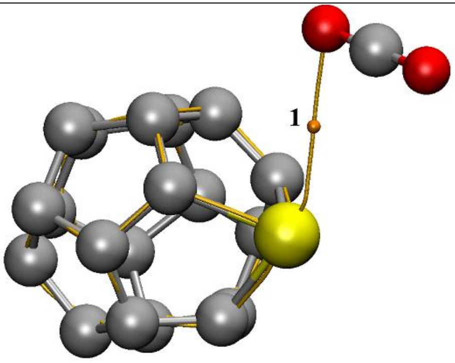
</td><td>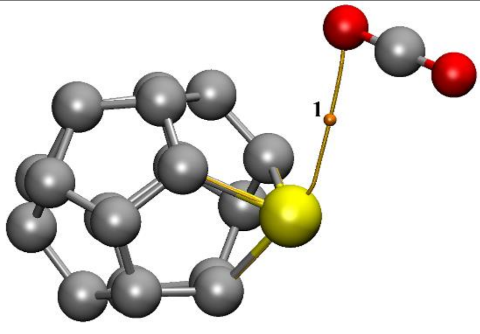
</td></tr>
<tr><td colspan="2">RDG</td></tr>
</table>

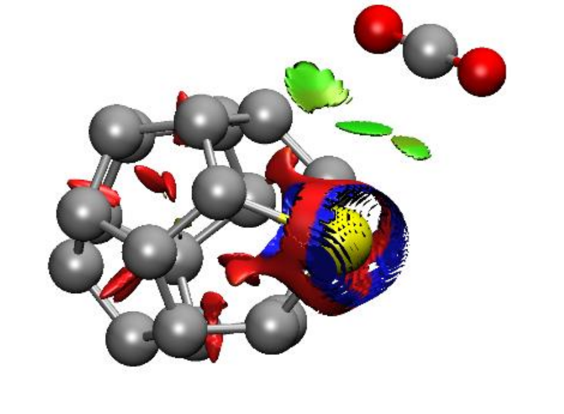

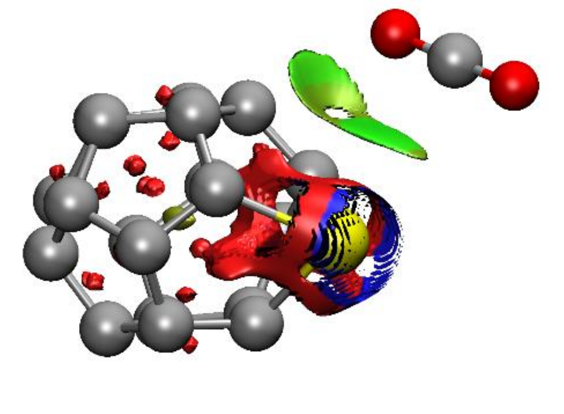

Scatter graph

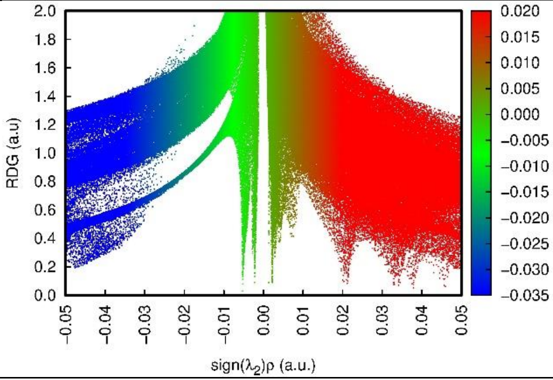

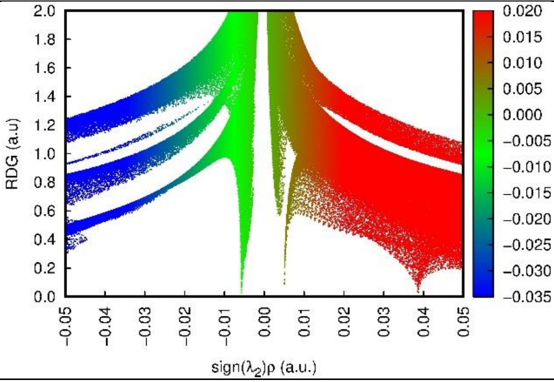

**C_{19}Si-N_2**<br>$F = 0.001\ a.u.$<br>$E_{ads}(BSSE) = -0.04514641\ eV$	**C_{19}Si-N_2**<br>$F = 0.001\ a.u.$<br>$E_{ads}(BSSE) = \ -0.04598611\ eV$

QTAIM

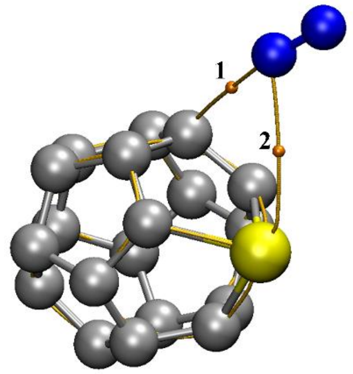

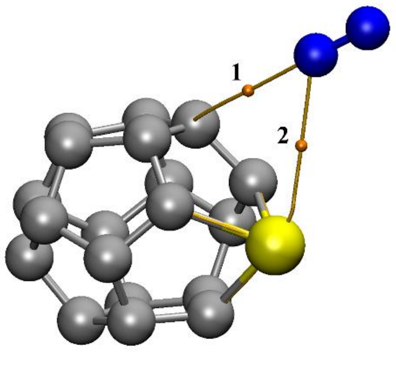

RDG

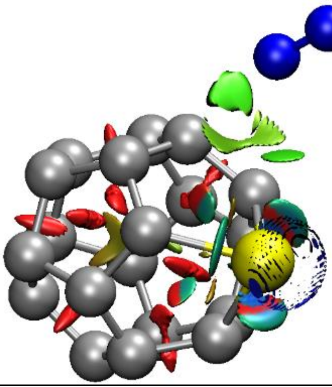

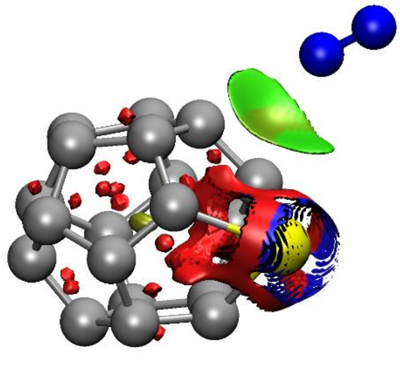

Scatter graph

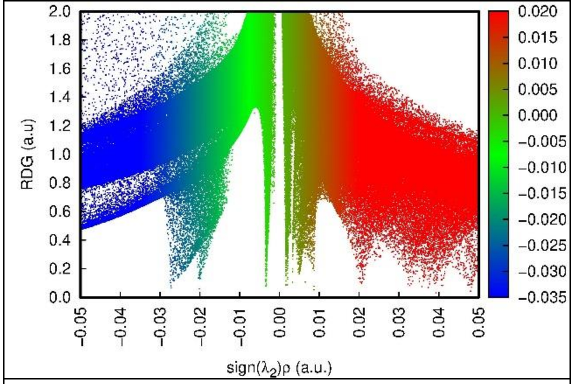	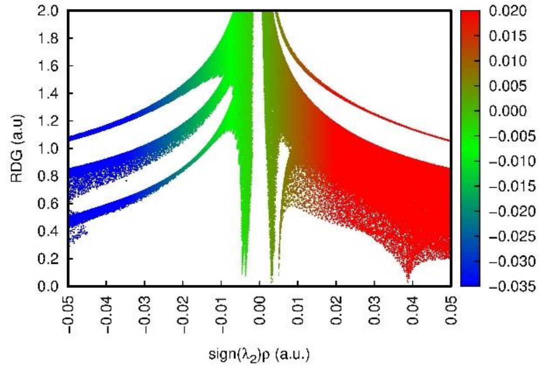
**C_{19}Si-CO**<br>$F = 0.005\ a.u.$<br>$E_{ads}(BSSE) = -0.95684190\ eV$	**C_{19}Si-CO**<br>$F = 0.005\ a.u.$<br>$E_{ads}(BSSE) = -1.00925177\ eV$
QTAIM	
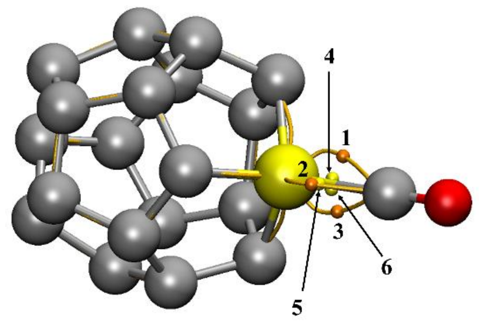	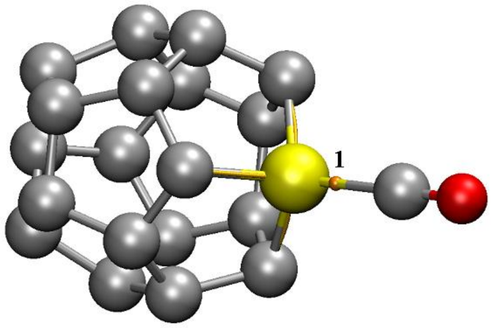
RDG	
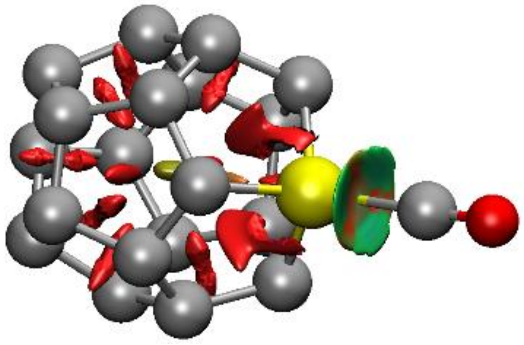	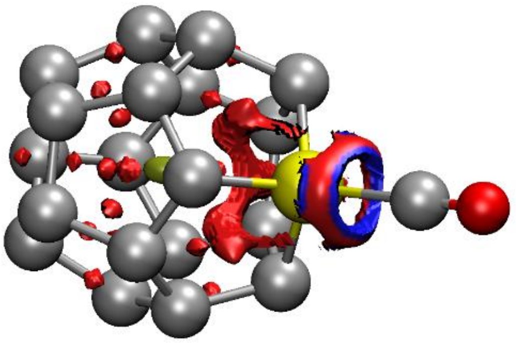
Scatter graph	
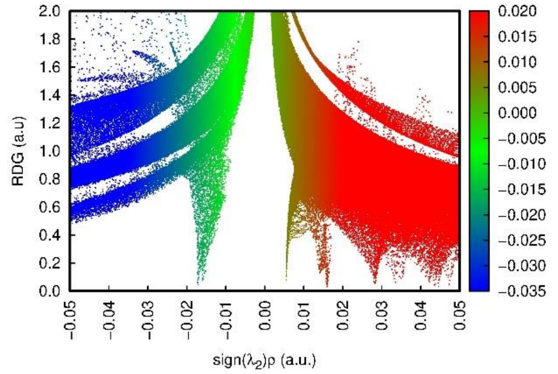	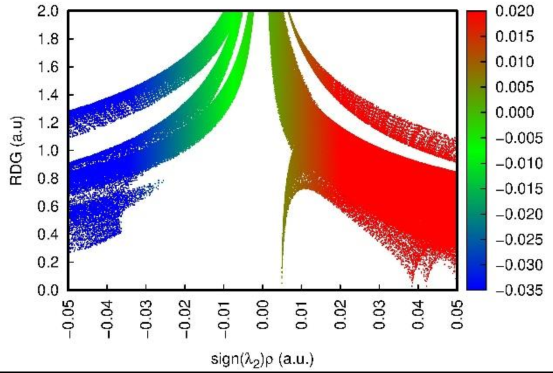
**C_{19}Si-CO_2**<br>$F = 0.005\ a.u.$<br>$E_{ads}(BSSE) = -0.08494219\ eV$	**C_{19}Si-CO_2**<br>$F = 0.005\ a.u.$<br>$E_{ads}(BSSE) = -0.09138179\ eV$
QTAIM	

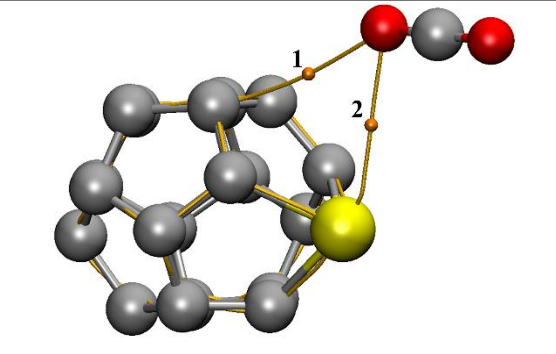	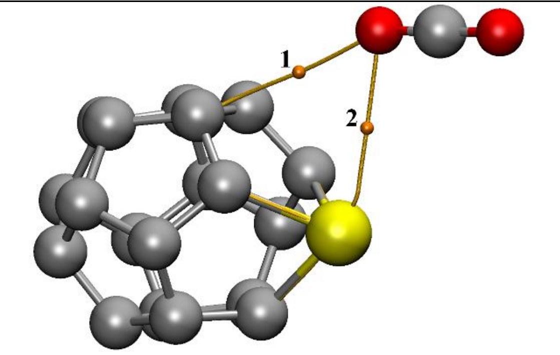
RDG	
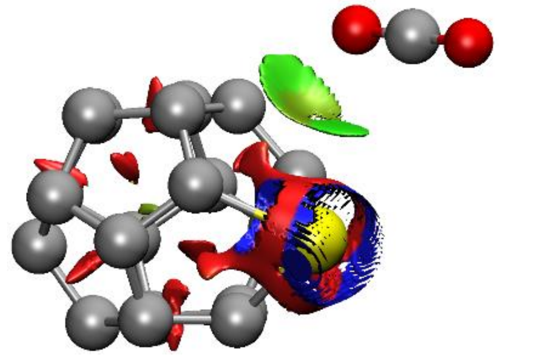	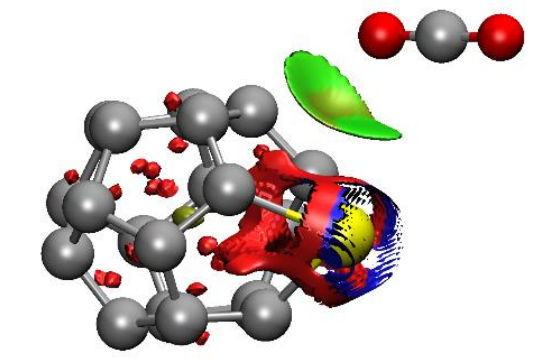
Scatter graph	
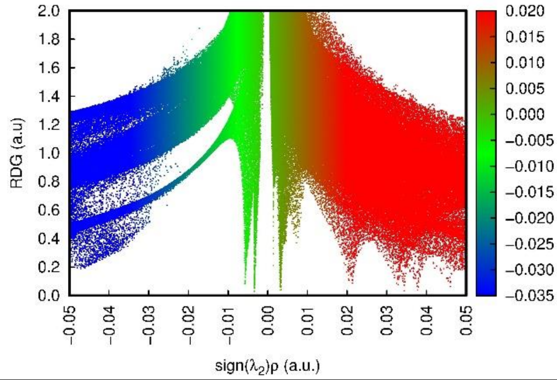	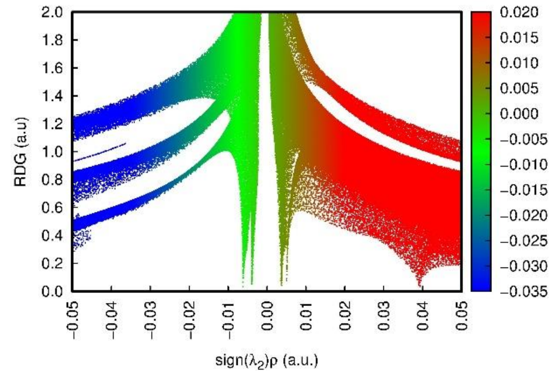
**$C_{19}Si$-N_2**<br>$F = 0.005\ a.u.$<br>$E_{ads}(BSSE) = -0.05377844\ eV$	**$C_{19}Si$-N_2**<br>$F = 0.005\ a.u.$<br>$E_{ads}(BSSE) = -0.05636742\ eV$
QTAIM	
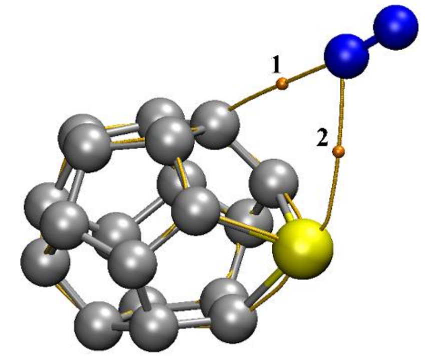	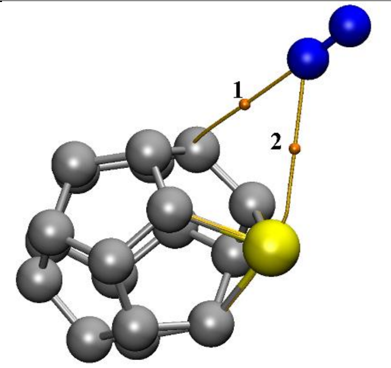
RDG	

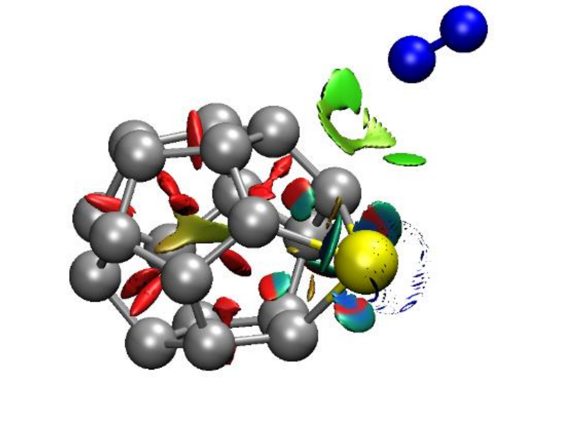	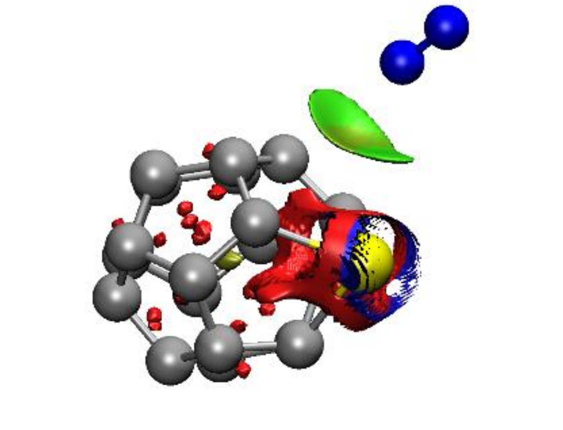
Scatter graph	
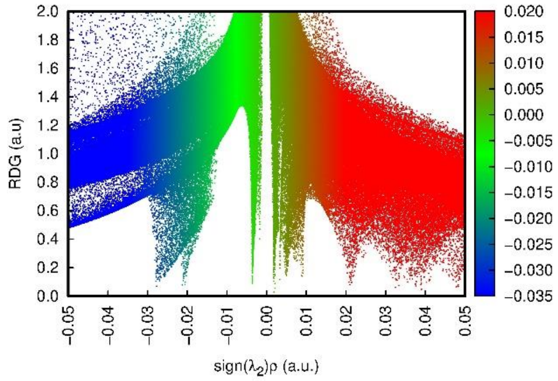	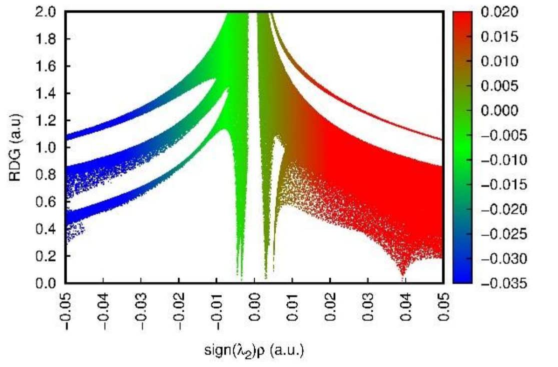
**C_{19}Si-CO**<br>$F = 0.010\ a.u.$<br>$E_{ads}(BSSE) = -1.26088602\ eV$	**C_{19}Si-CO**<br>$F = 0.010\ a.u.$<br>$E_{ads}(BSSE) = -1.32802545\ eV$
QTAIM	
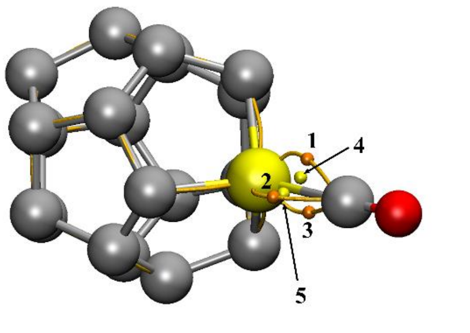	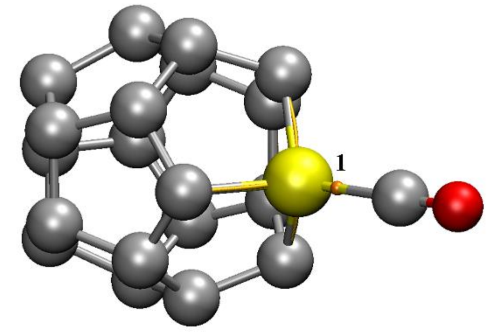
RDG	
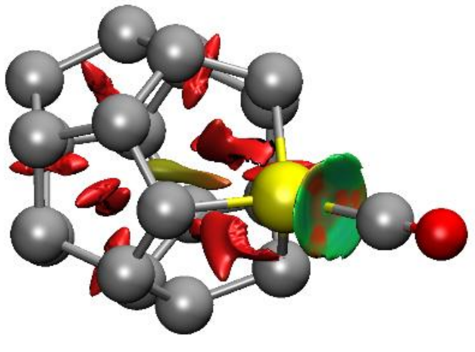	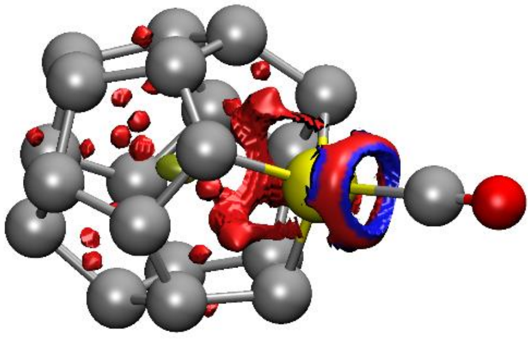
Scatter graph	

<table>
<tr><td>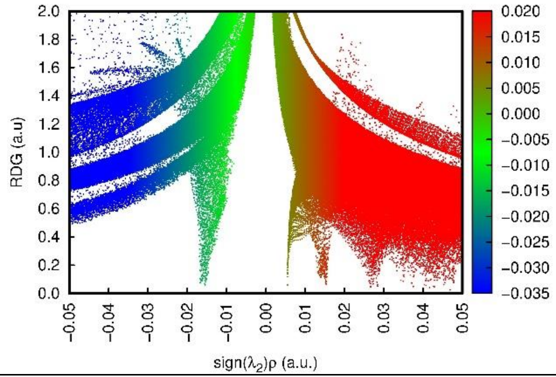
</td>
<td>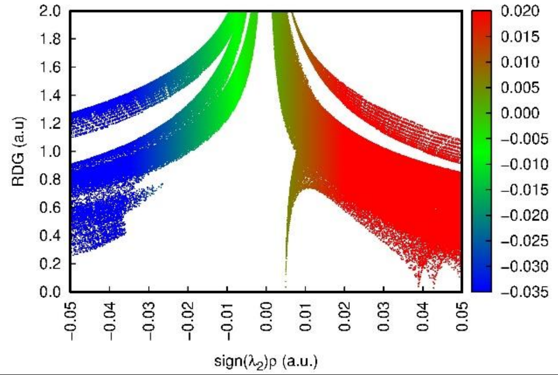
</td></tr>
<tr><td>$C_{19}Si$-CO_2
$F = 0.010\ a.u.$
$E_{ads}(BSSE) = -0.10993079\ eV$</td>
<td>$C_{19}Si$-CO_2
$F = 0.010\ a.u.$
$E_{ads}(BSSE) = -0.12354919\ eV$</td></tr>
<tr><td colspan="2">QTAIM</td></tr>
<tr><td>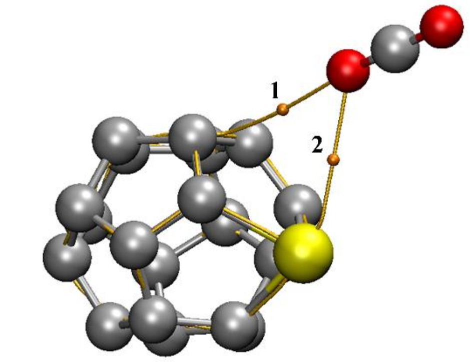
</td>
<td>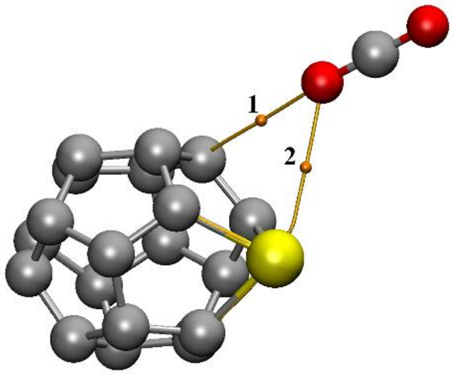
</td></tr>
<tr><td colspan="2">RDG</td></tr>
<tr><td>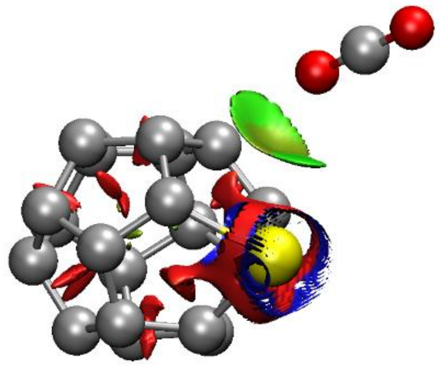</td><td>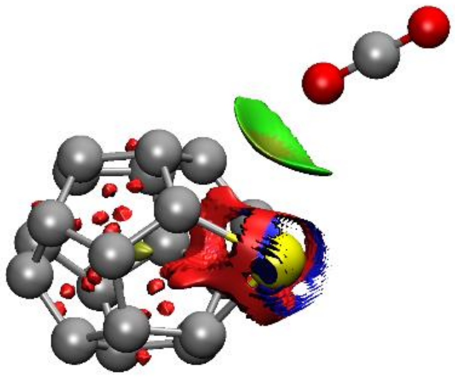</td></tr>
<tr><td colspan="2">Scatter graph</td></tr>
<tr><td>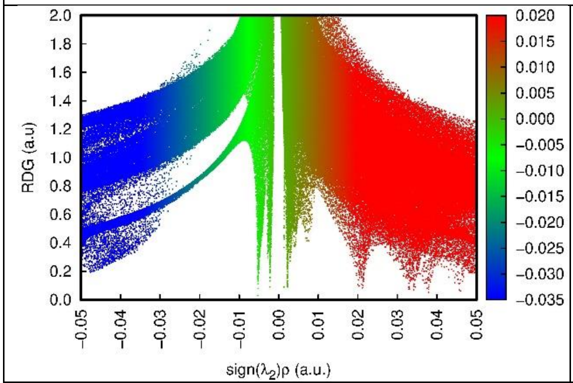
</td>
<td>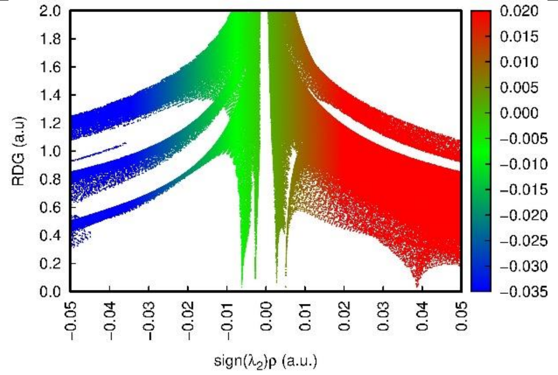
</td></tr>
<tr><td>$C_{19}Si$-N_2
$F = 0.010\ a.u.$</td>
<td>$C_{19}Si$-N_2
$F = 0.010\ a.u.$</td></tr>
</table>

$E_{ads}(BSSE) = -0.07166555\ eV$	$E_{ads}(BSSE) = -0.07659200\ eV$
QTAIM	
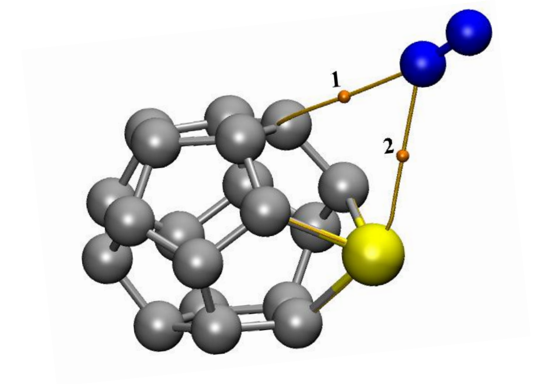	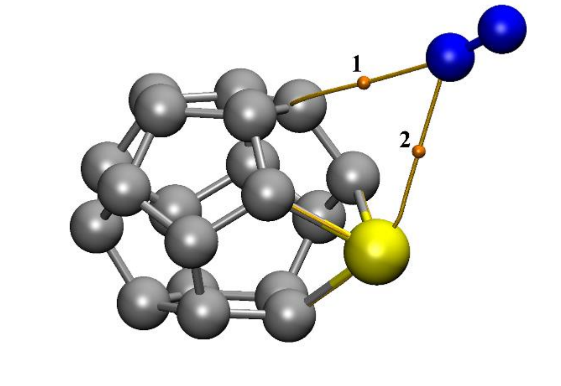
RDG	
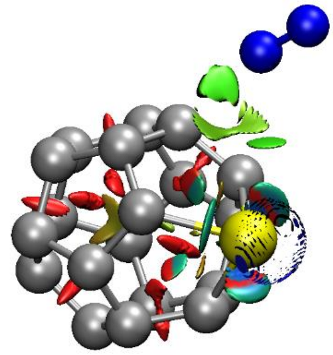	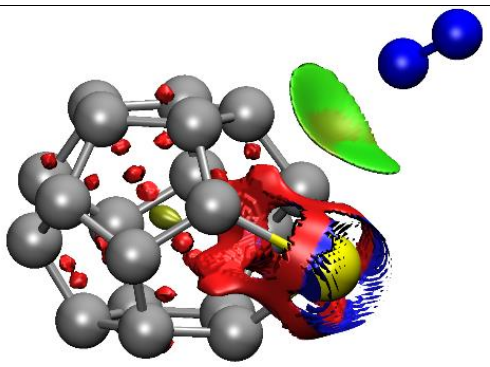
Scatter graph	
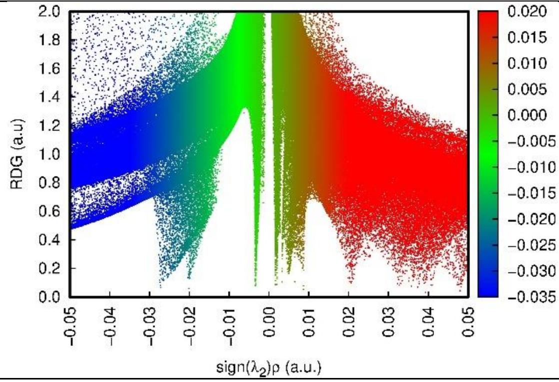	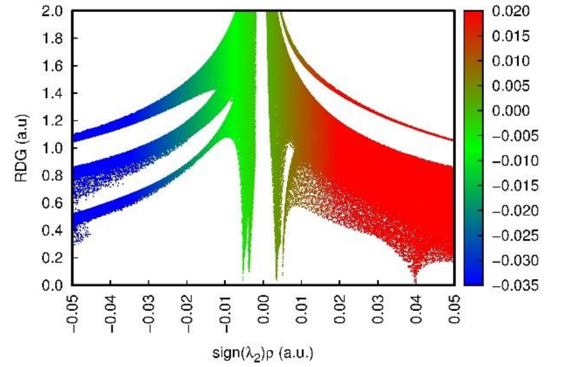
**C19Si-CO**<br>$F = 0.015\ a.u.$<br>$E_{ads}(BSSE) = -1.60888130\ eV$	**C19Si-CO**<br>$F = 0.015\ a.u.$<br>$E_{ads}(BSSE) = -1.69386335\ eV$
QTAIM	
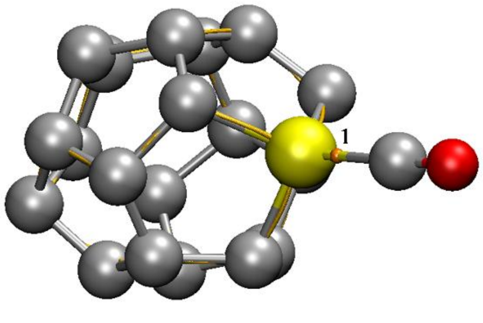	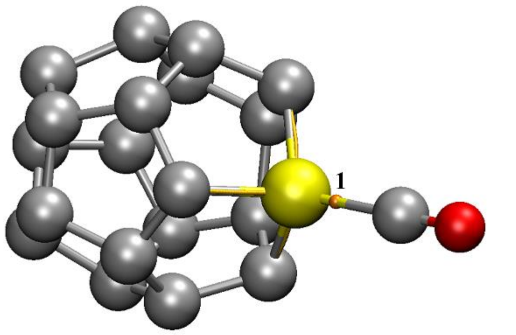
RDG	

<table>
<tr><td>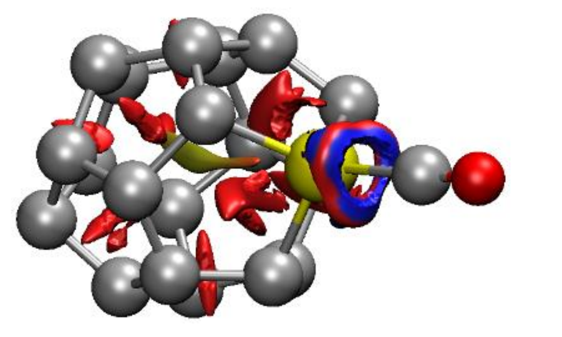</td><td>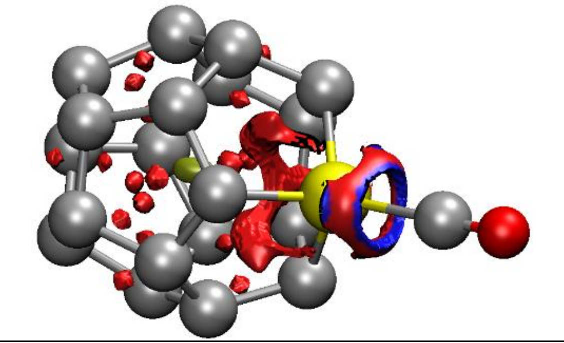</td></tr>
<tr><td colspan="2">Scatter graph</td></tr>
<tr><td>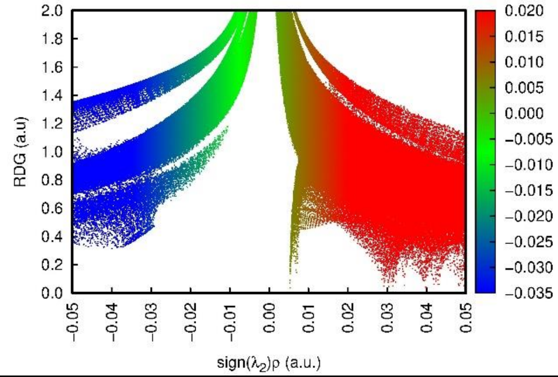
</td><td>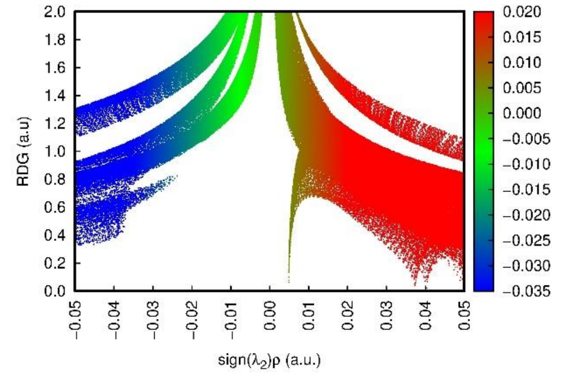
</td></tr>
<tr><td>C_{19}Si-CO_2
$F = 0.015\ a.u.$
$E_{ads}(BSSE) = -0.16062570\ eV$</td><td>$C_{19}$Si-$CO_2$
$F = 0.015\ a.u.$
$E_{ads}(BSSE) = -0.19373070\ eV$</td></tr>
<tr><td colspan="2">QTAIM</td></tr>
<tr><td>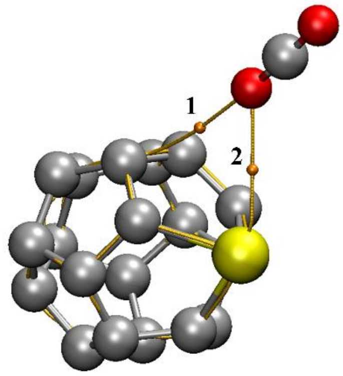
</td><td>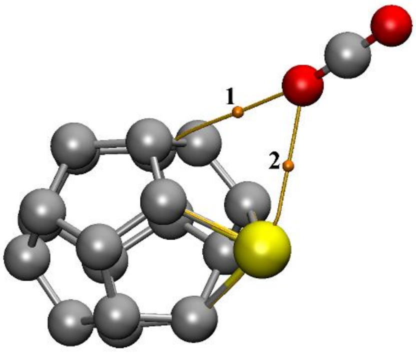
</td></tr>
<tr><td colspan="2">RDG</td></tr>
<tr><td>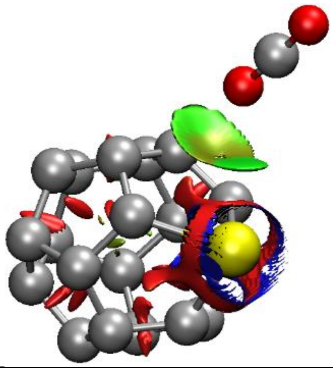</td><td>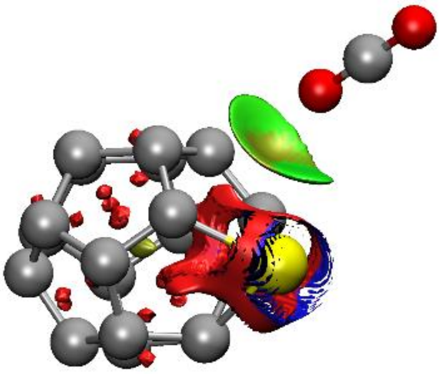</td></tr>
<tr><td colspan="2">Scatter graph</td></tr>
</table>

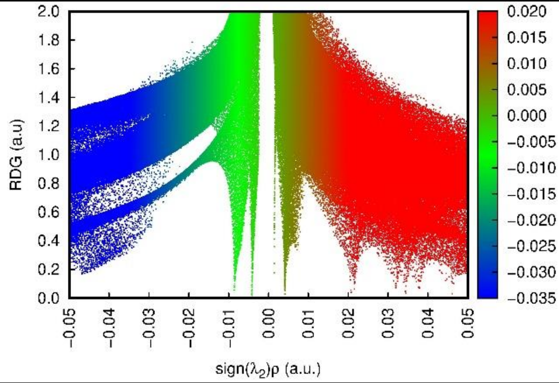

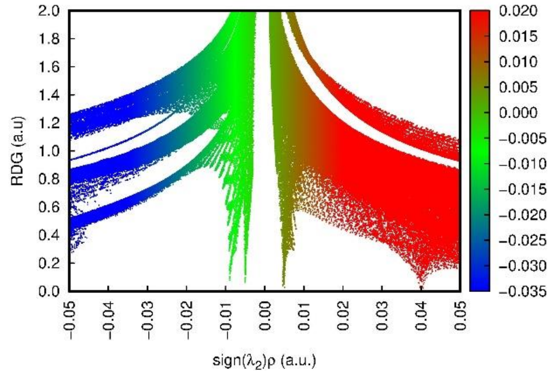

C_{19}Si-N_2
$F = 0.015\ a.u.$
$E_{ads}(BSSE) = -0.09723814\ eV$

C_{19}Si-N_2
$F = 0.015\ a.u.$
$E_{ads}(BSSE) = -0.11392979\ eV$

QTAIM

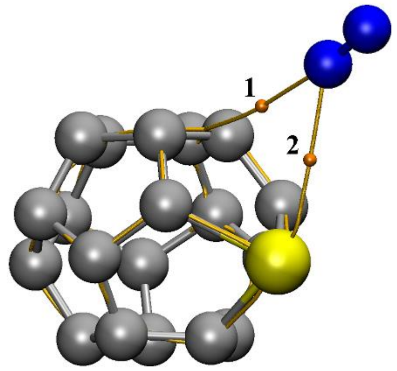

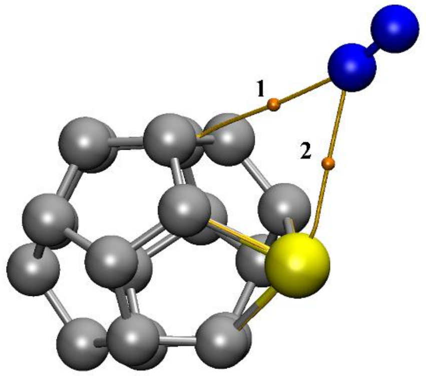

RDG

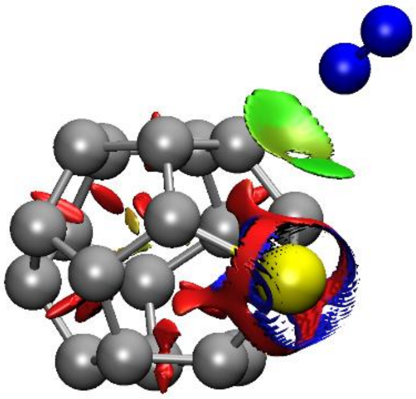

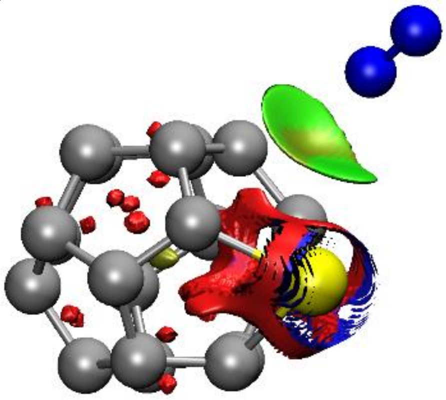

Scatter graph

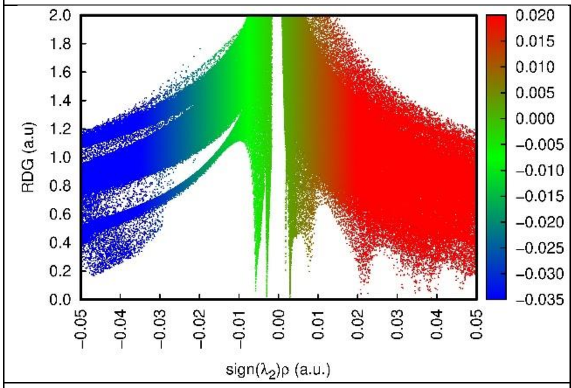

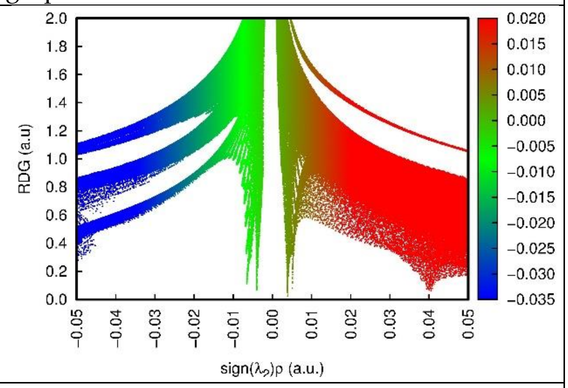

C_{19}Si-CO
$F = 0.020\ a.u.$
$E_{ads}(BSSE) = -1.98886525\ eV$

C_{19}Si-CO
$F = 0.020\ a.u.$
$E_{ads}(BSSE) = -2.10539484\ eV$

QTAIM	
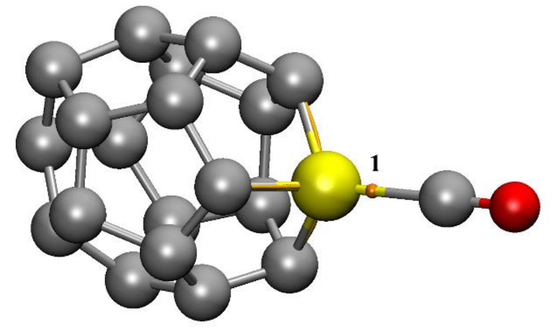 	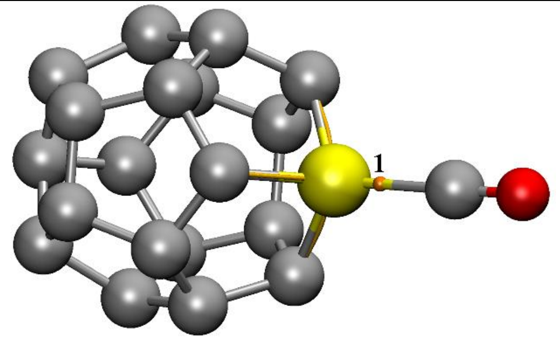
RDG	
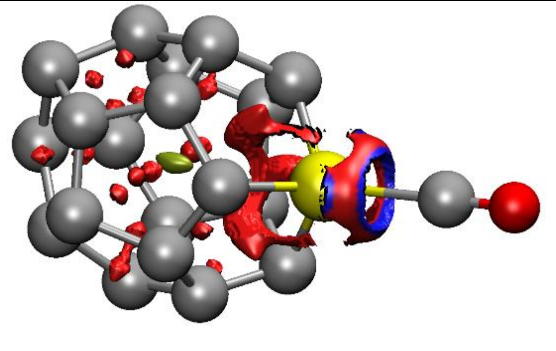	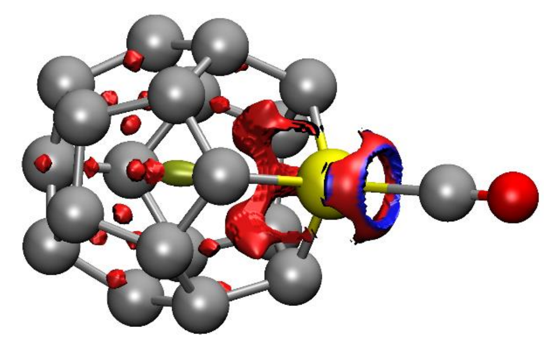
Scatter graph	
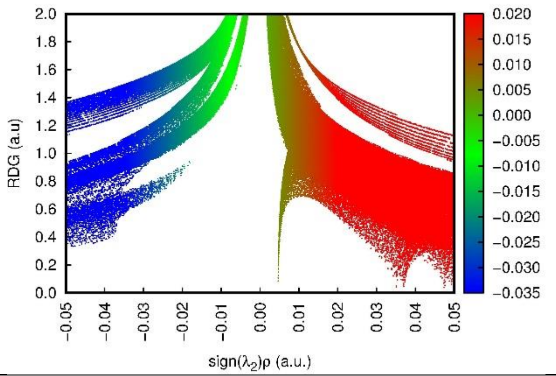 	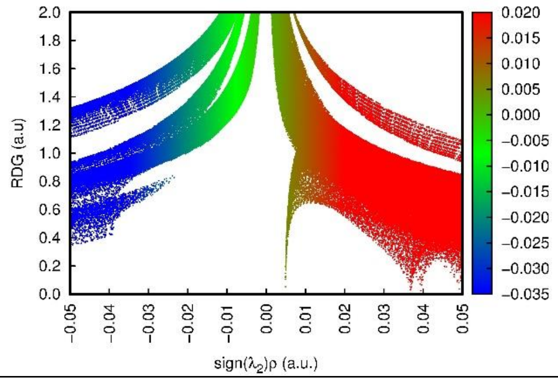
**C_{19}Si-CO_2**<br>$F = 0.020\ a.u.$<br>$E_{ads}(BSSE) = -0.23431939\ eV$	**C_{19}Si-CO_2**<br>$F = 0.020\ a.u.$<br>$E_{ads}(BSSE) = -0.27245444\ eV$
QTAIM	
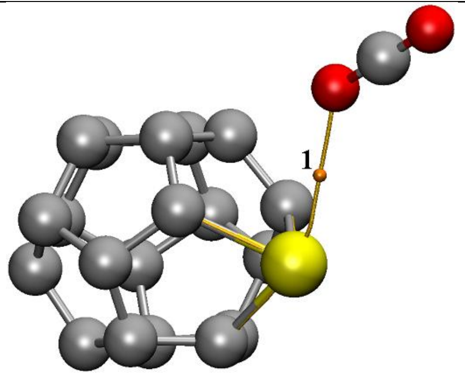 	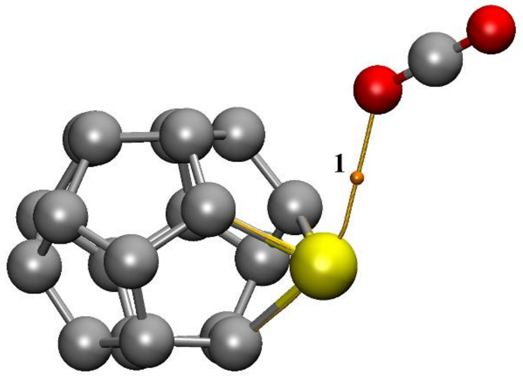
RDG	

<table>
<tr><td>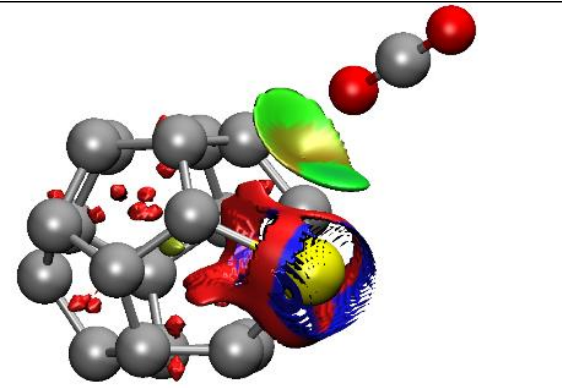</td><td>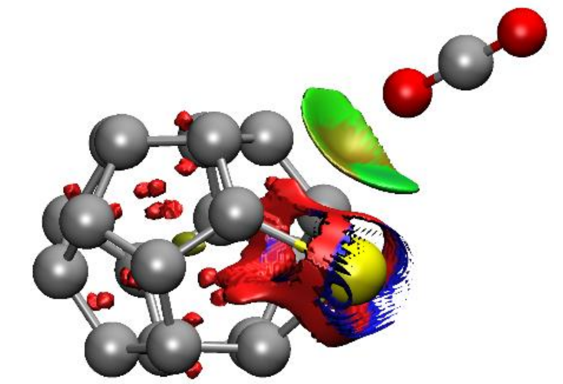</td></tr>
<tr><td colspan="2">Scatter graph</td></tr>
<tr><td>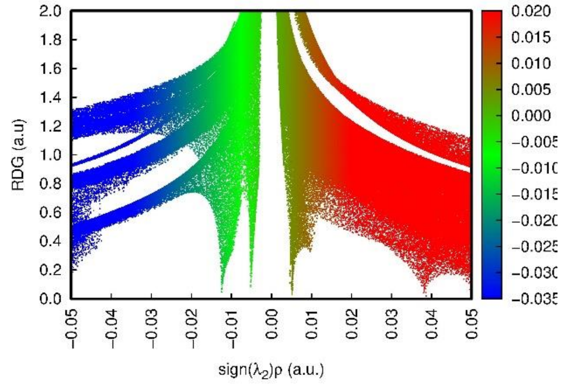
</td><td>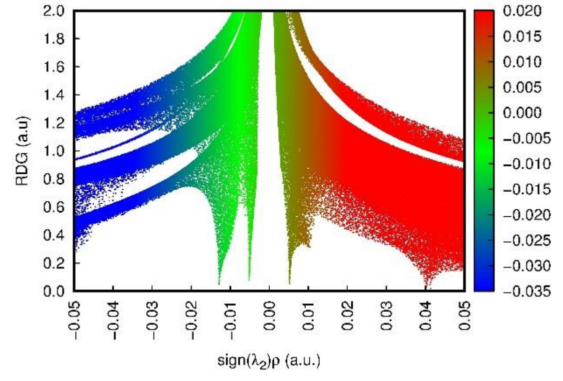
</td></tr>
<tr><td>$C_{19}Si$-N_2
$F = 0.020\ a.u.$
$E_{ads}(BSSE) = -0.13186518\ eV$</td><td>$C_{19}Si$-$N_2$
$F = 0.020\ a.u.$
$E_{ads}(BSSE) = -0.14614638\ eV$</td></tr>
<tr><td colspan="2">QTAIM</td></tr>
<tr><td>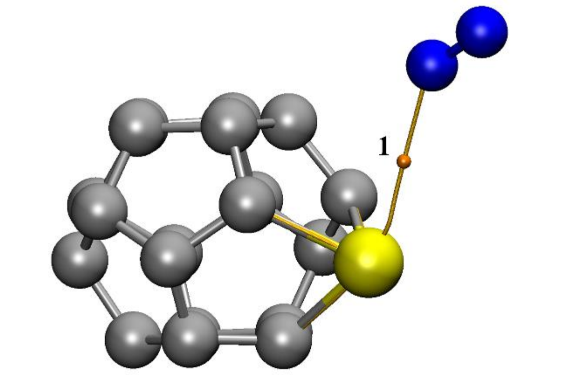
</td><td>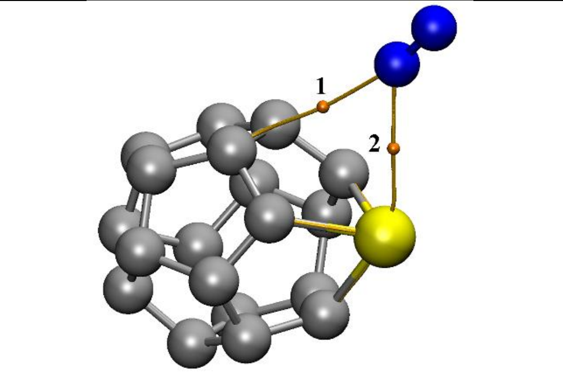
</td></tr>
<tr><td colspan="2">RDG</td></tr>
<tr><td>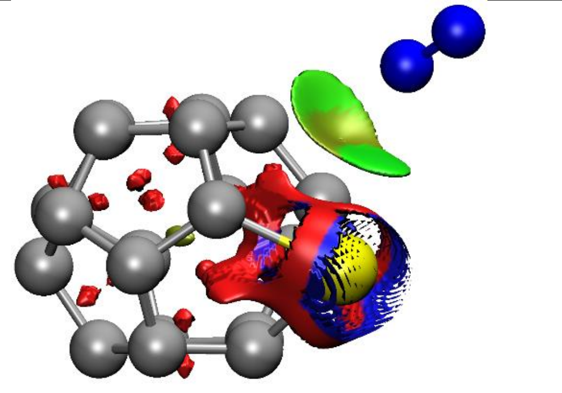</td><td>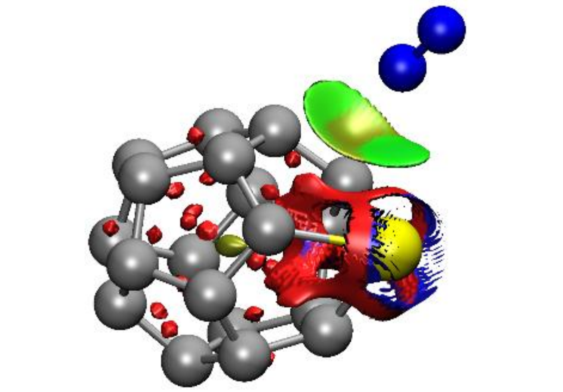</td></tr>
<tr><td colspan="2">Scatter graph</td></tr>
</table>

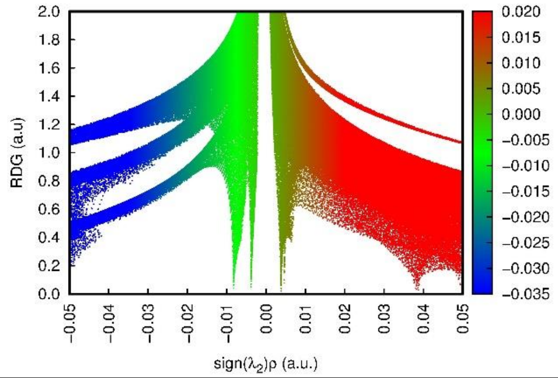

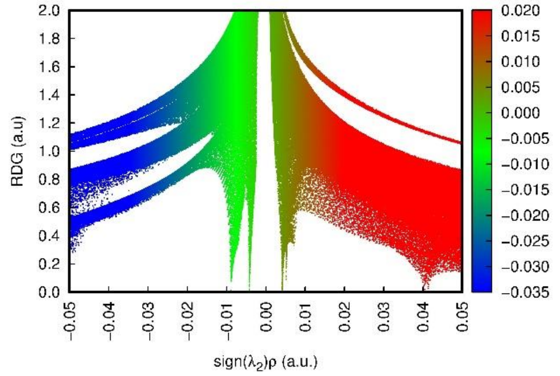

**C_{19}Si-CO**<br>$F = 0.025\ a.u.$<br>$E_{ads}(BSSE) = -2.40028075 eV$	**C_{19}Si-CO**<br>$F = 0.025\ a.u.$<br>$E_{ads}(BSSE) = -2.57870530\ eV$

QTAIM

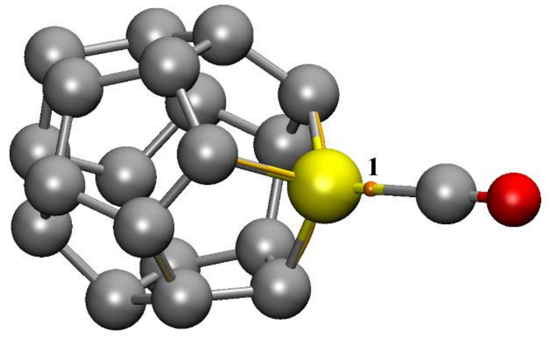

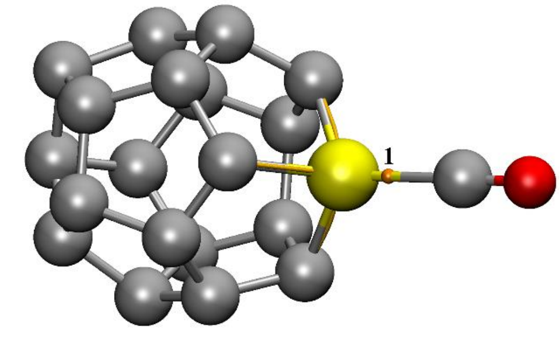

RDG

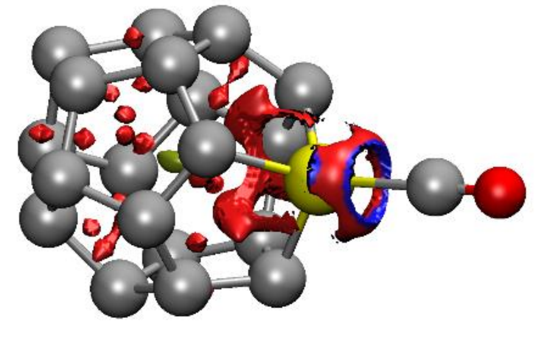

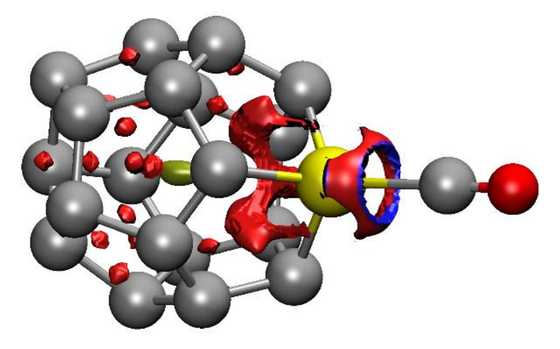

Scatter graph

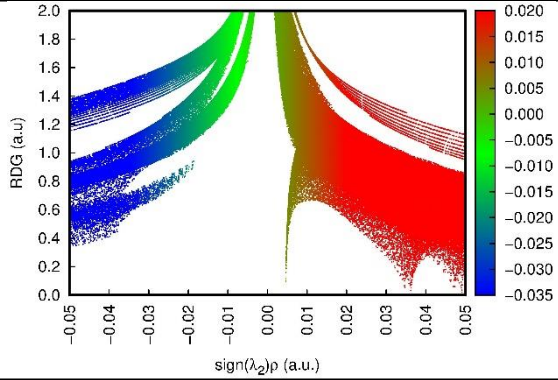

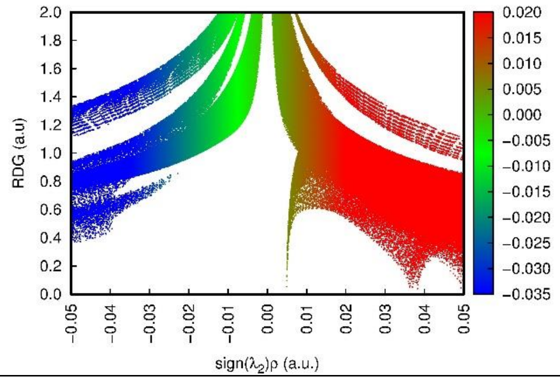

**C_{19}Si-CO_2**<br>$F = 0.025\ a.u.$<br>$E_{ads}(BSSE) = -0.33903162\ eV$	**C_{19}Si-CO_2**<br>$F = 0.025\ a.u.$<br>$E_{ads}(BSSE) = -0.40174203 eV$

QTAIM

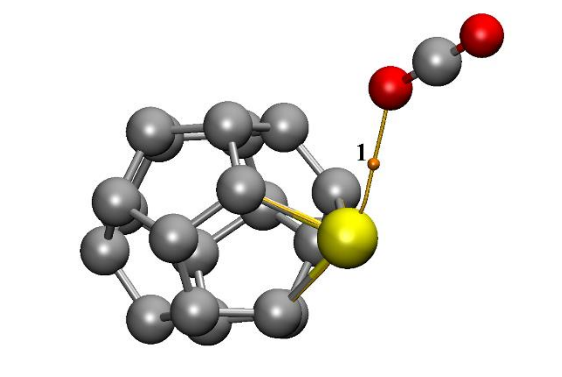	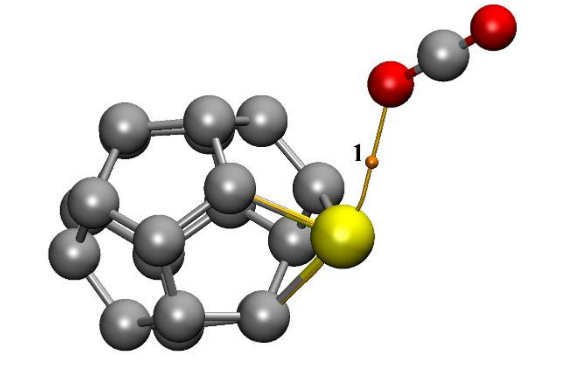
RDG	
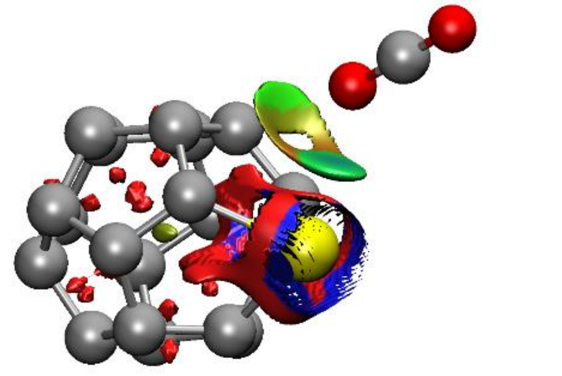	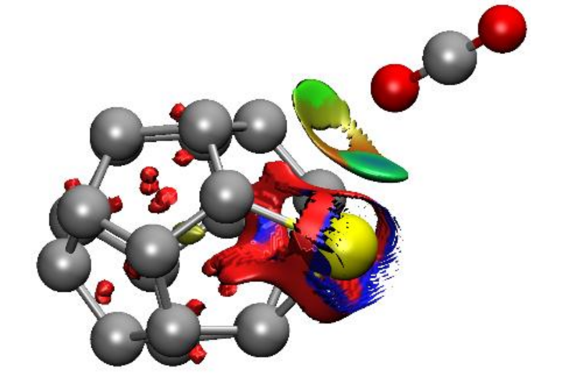
Scatter graph	
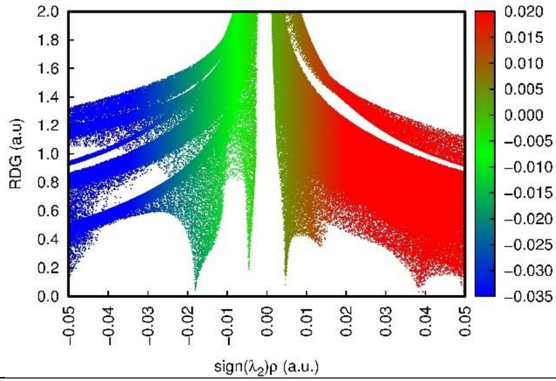	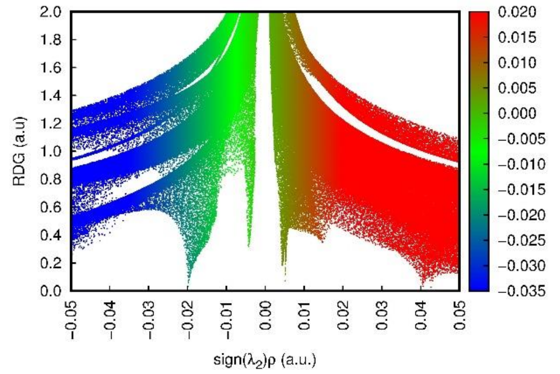
**C_{19}Si-N_2**<br>$F = 0.025\ a.u.$<br>$E_{ads}(BSSE) = -0.17736559\ eV$	**C_{19}Si-N_2**<br>$F = 0.025\ a.u.$<br>$E_{ads}(BSSE) = \ -0.20140237eV$
QTAIM	
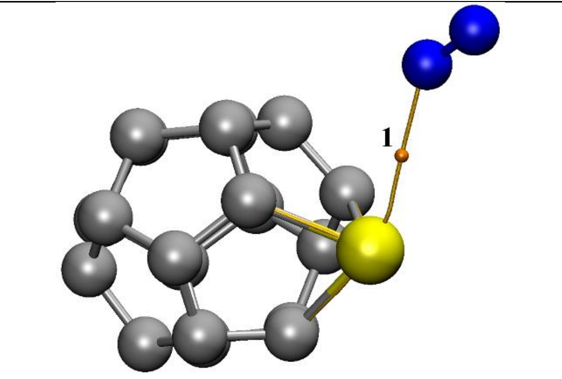	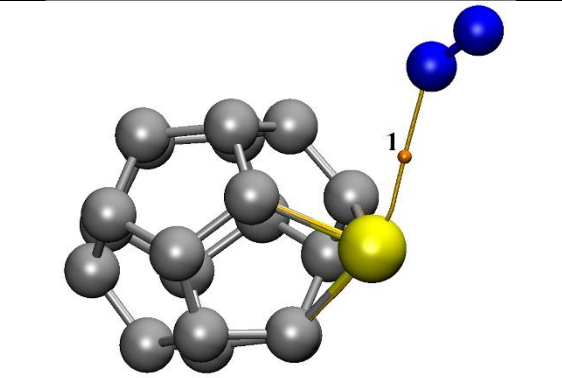
RDG	

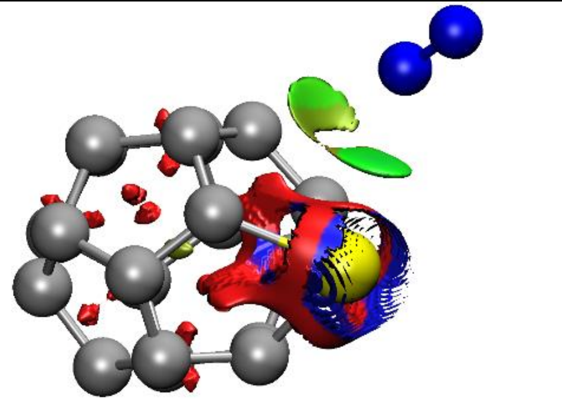

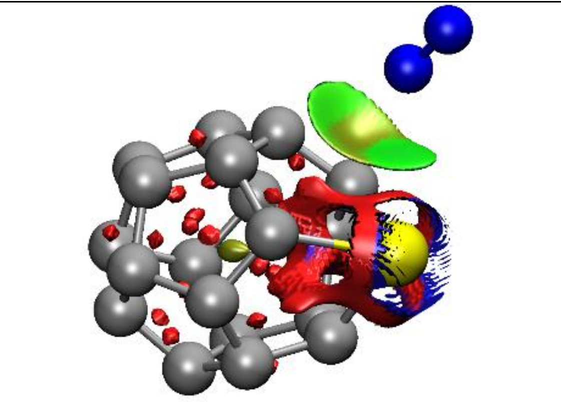

Scatter graph

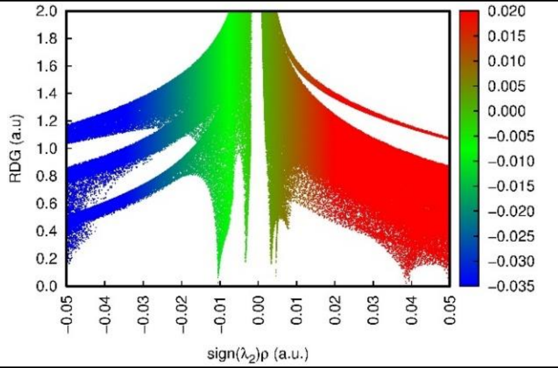

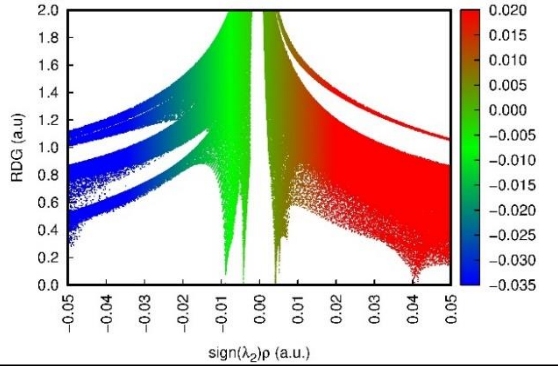

Supplement: Supplementary file 1 [file molecules-27-02968-s001.zip › molecules-1641131-supplementary.pdf]
